# ShcD adaptor protein drives invasion of triple negative breast cancer cells by aberrant activation of EGFR signaling

**DOI:** 10.1002/1878-0261.70022

**Published:** 2025-03-28

**Authors:** Hayley R. Lau, Hayley S. Smith, Begüm Alural, Claire E. Martin, Laura A. New, Manali Tilak, Sara L. Banerjee, Hannah N. Robeson, Nicolas Bisson, Anne‐Claude Gingras, Jasmin Lalonde, Nina Jones

**Affiliations:** ^1^ Department of Molecular and Cellular Biology University of Guelph Canada; ^2^ Lunenfeld‐Tanenbaum Research Institute Sinai Health System Toronto Canada; ^3^ Cancer Research Centre Quebec Network for Research on Protein Function, Engineering, and Applications (PROTEO) and Centre Hospitalier Universitaire de Québec Research Centre‐Université Laval Québec City Canada

**Keywords:** breast cancer, cell signaling, EGFR, invasion, ShcD/Shc4

## Abstract

Triple‐negative breast cancer (TNBC) is highly metastatic and presents clinical challenges given the lack of targeted therapies. Here, we report that the ShcD phosphotyrosine adaptor protein is upregulated in TNBC, and its expression correlates with overall reduced patient survival and decreased response to chemotherapy. In human breast cancer cells, we demonstrate that ShcD expression promotes cell invasion and reduces adhesion, and that these effects are abrogated by mutating the ShcD phosphotyrosine binding (PTB) domain. Similarly, in a three‐dimensional assembloid model, ShcD‐expressing spheroids derived from brain metastatic TNBC cells show enhanced infiltration into cerebral organoids. Using a proteomic screen for ShcD binding partners, we identify multiple components of epidermal growth factor receptor (EGFR) signaling and confirm these interactions with ShcD but not the PTB mutant. Interestingly, the ShcD interactome correlates with EGFR tyrosine kinase inhibitor resistance, in line with our findings that ShcD overexpression results in hyperphosphorylation of EGFR while ShcD knockout or PTB mutation reverts this response. Lastly, pharmacological inhibition of the ShcD PTB domain using indomethacin in TNBC cells decreases EGFR binding and hyperphosphorylation and reduces cell invasion. Altogether, our results identify ShcD as a potential contributor to metastasis in TNBC, and they provide a molecular basis for clinical targeting of adaptor proteins.

## Graphical abstract

We identified adaptor protein ShcD as upregulated in triple‐negative breast cancer and found its expression to be correlated with reduced patient survival and increased invasion in cell models. Using a proteomic screen, we identified novel ShcD binding partners involved in EGFR signaling pathways. Finally, inactivation of ShcD phosphotyrosine‐binding domains or treatment with the PTB‐binding small‐molecule drug indomethacin inhibited tumour cell invasion.

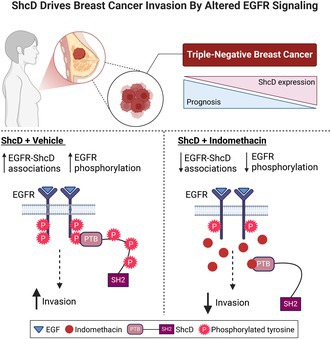

**Abbreviations**
2D: 2‐dimensional
3D: 3‐dimensional
ACC: adrenocortical carcinoma
AP‐MS: affinity‐purification mass spectrometry
BC: breast cancer
BLCA: bladder urothelial carcinoma
BRCA: breast invasive carcinoma
CESC: cervical squamous cell carcinoma and endocervical adenocarcinoma
CHOL: cholangiocarcinoma
COAD: colon adenocarcinoma
DLBC: lymphoid neoplasm diffuse large B‐cell lymphoma
EGF: epidermal growth factor
EGFR: epidermal growth factor receptor
ESCA: esophageal carcinoma
GAB: grb2‐associated scaffold proteins
GBM: glioblastoma multiforme
GFP: green fluorescent protein
HNSC: head and neck squamous cell carcinoma
IB: immunoblotting
IP: immunoprecipitation
iPSC: induced pluripotent stem cell
KICH: kidney chromophobe renal cell carcinoma
KIRC: kidney renal clear cell carcinoma
KIRP: kidney renal papillary cell carcinoma
KO: knockout
LAML: acute myeloid leukemia
LGG: brain lower grade glioma
LIHC: liver hepatocellular carcinoma
LUAD: lung adenocarcinoma
LUSC: lung squamous cell carcinoma
MESO: mesothelioma
NT: non‐targeting
OE: overexpression
OV: ovarian cancer
PAAD: pancreatic adenocarcinoma
PCPG: pheochromocytoma and paraganglioma
PI3K: phosphoinositide 3‐kinase
PRAD: prostate adenocarcinoma
PTB: phosphotyrosine binding
pY: phosphotyrosine
READ: rectum adenocarcinoma
RTK: receptor tyrosine kinase
SARC: sarcoma
SD: standard deviation
SH2: src homology 2
SKCM: skin cutaneous melanoma
STAD: stomach adenoma carcinoma
TCGA: The Cancer Genome Atlas
TCGT: tenosynovial giant cell tumor
THCA: thyroid carcinoma
THYM: thymoma
TNBC: triple‐negative breast cancer
UCEC: uterine corpus endometrial carcinoma
UCS: uterine carcinosarcoma
UVM: uveal melanoma
WT: wildtype

## 1 Introduction

Metastatic breast cancers are responsible for the highest occurrence of cancer deaths in women worldwide, and they remain largely incurable. Driven by a series of deregulated signaling events, malignant cells acquire invasive properties, which facilitate their spread to other sites, including the lung, liver, bone, and brain [1]. Notably, triple‐negative breast cancer (TNBC) is a particularly aggressive subtype and correlates with poor prognosis owing to the lack of defined molecular targets [2]. Identification of new therapeutic entry points is therefore essential to control metastasis and improve outcomes in this devastating disease.

Aberrant activation of receptor tyrosine kinases (RTKs) such as the epidermal growth factor receptor (EGFR) is a well‐characterized contributor to cancer cell invasion and metastasis. Following stimulation with its cognate ligand, epidermal growth factor (EGF), phosphorylated EGFR can engage and activate Grb2‐associated binding (GAB) scaffold proteins, inducing recruitment of the p85 regulatory subunit of phosphatidylinositol‐3 kinase (PI3K), ultimately leading to activation of Akt [3]. Akt proteins control many aspects of breast cancer development and display both pro‐ and antimetastatic functions in disease progression [4, 5, 6]. Although EGFR is not a central receptor in defining breast cancer subtypes, it is overexpressed in up to 78% of breast cancer cases [7] and is associated with poor clinical outcomes and low survival rates [8, 9]. Given the correlation between elevated EGFR and the increased metastatic potential of breast cancer cells [10, 11], many anti‐EGFR therapies have been developed. However, these approaches have shown minimal efficacy in clinical trials [12], thereby highlighting a need to investigate novel mechanisms driving altered EGFR signaling.

ShcD (SHC4/RALP) is a member of the Shc family of adaptor proteins characterized by an amino‐terminal phosphotyrosine binding (PTB) domain and a carboxy‐terminal Src‐homology 2 (SH2) domain, which both mediate binding to phosphotyrosine residues on active RTKs, including EGFR [13]. Shc adaptors include four distinct paralogs, ShcA‐D, which are essential for coupling upstream inputs with downstream signaling pathways such as Erk and PI3K/Akt via the phosphorylation of conserved central tyrosine residues. We previously uncovered that ShcD induces ligand‐independent EGFR hyperphosphorylation [14], which may have clinical implications in the context of gliomas, where ShcD upregulation is correlated with EGFR phosphorylation [14]. Moreover, we recently demonstrated that ShcD also induces hyperphosphorylation of the Tie2 RTK and promotes glioma infiltration into human cerebral organoids [15]. In addition to gliomas, ShcD upregulation has also been detected in late‐stage human melanomas, where it appears to be a key molecule in driving metastatic progression [16, 17].

Now, in a pan‐cancer analysis of ShcD expression, we identify its upregulation in human breast cancers, most notably in TNBC, where it correlates with reduced overall patient survival. In MDA‐MB‐231 TNBC cells, ShcD expression results in increased cell invasion and reduced adhesion. Using an AP‐MS (affinity‐purification mass spectrometry) screen in TNBC cells, we identify phosphotyrosine‐dependent interactions between ShcD and EGFR complex components, among other novel interactors. Mechanistically, we demonstrate that ShcD enhances EGF‐stimulated EGFR phosphorylation but reduces Akt phosphorylation and show that these responses are lost upon mutation of ShcD PTB/SH2 domains or ShcD knockout. These events are recapitulated in the brain metastatic variant MDA‐MB‐231BR cell line where ShcD overexpression promotes heightened cell infiltration into a human brain assembloid model. Lastly, we show that targeted blockade of the Shc PTB domain with indomethacin reduces ShcD‐EGFR binding, EGFR hyperphosphorylation, and cell invasion. These results suggest a link between ShcD‐induced EGFR hyperphosphorylation and cell invasion and position ShcD as a putative target to limit breast cancer metastasis.

## 2 Materials and methods

### 2.1 Plasmids

Full‐length cDNAs encoding wild‐type (WT) human ShcD (BC033907.1) or versions with mutations in the PTB domain (R315Q, denoted PTB*), SH2 domain (R548K, denoted SH2*) or both (denoted ShcD PTB*/SH2*) were previously cloned into the pcDNA3 vector (Invitrogen) with a C‐terminal triple FLAG epitope [14, 18] and subcloned for stable overexpression using the sleeping beauty transposon system. *SfiI* restriction sites were added to each end of each cDNA, including the C‐terminal triple FLAG tag, and cloned into the pSBbi‐RP plasmid, a gift from Eric Kowarz (Addgene; Plasmid #60513) [19], resulting in plasmids that express ShcD‐FLAG and red fluorescent protein separately. We also generated a control 3×FLAG‐pSBbi‐RP plasmid.

### 2.2 Antibodies

The following antibodies were obtained commercially and used for immunoblotting analysis at the indicated dilutions (prepared in 1× TBST): mouse anti‐FLAG clone M2 at 1:1000 (Sigma‐Aldrich, Burlington, MA, USA; Cat. #F3165), rabbit anti‐Akt at 1:1000 (Cell Signaling Technology, Danvers, MA, USA; Clone C67E7; Cat. #4691S), rabbit anti‐p‐Akt (S473) at 1:1000 (Cell Signaling Technology; Clone D9E Cat. #4060S), rabbit anti‐pShc (Tyr‐239/240) at 1:1000 (Cell Signaling Technology; Cat. #2434S), rabbit anti‐EGFR at 1:500 (Santa Cruz, Dallas, TX, USA; Clone B2610 Cat. #sc‐03;), rabbit anti‐EGFR at 1:1000 (Cell Signaling Technology, Cat. #2232S), rabbit anti‐Erk at 1:2000 (Cell Signaling Technology; Clone 137F5 Cat. #4695S), rabbit anti‐pErk (T202/Y204) at 1:1000 (Cell Signaling Technology; Clone D13.14.4E Cat. #9101S), mouse anti‐pY at 1:1000 (clone 4G10, Sunnybrook Hospital Toronto, Antibody Core Facility), rabbit anti‐GAB1 at 1:500 (CT Upstate; Cat. #06‐579), rabbit anti‐p85‐PI3K (clone R1177; from A Klippel) [20], mouse anti‐ß‐actin at 1:2000 (Sigma‐Aldrich; Cat. #A1978), mouse anti‐ShcD (Developmental Studies Hybridoma Bank; Cat. #AFFN‐SHC4‐9E1) at 1:500, mouse anti‐ShcD (Sigma‐Aldrich; Clone 2F5 Cat. #WH0399694M1) at 1:500, and rabbit anti‐ShcD raised against the human ShcD N‐terminus [18] at 1:500. Horseradish peroxidase (HRP)‐conjugated goat anti‐rabbit and goat anti‐mouse (Bio‐Rad Laboratories, Hercules, CA, USA; Cat. #1706515 and #1706516 respectively) antibodies were used as secondary antibodies.

### 2.3 Cell culture and protein extraction

The human breast cancer cell lines used in this study, MDA‐MB‐231 (RRID:CVCL_0062), MCF‐7 (RRID:CVCL_0031), and MX‐1 (RRID:CVCL_4774), were originally sourced from the American‐Type Culture Collection (ATCC, Manassas, VA). Human cell lines MDA‐MB‐231, MDA‐MB‐231BR (brain metastatic derivative of MDA‐MB‐231) (RRID:CVCL_A0YY) [21], and MCF‐7 cells were grown in Dulbecco's modified Eagle medium (DMEM) (Wisent, Saint‐Jean‐Baptiste, Canada; Cat. #319‐015‐CL) supplemented with 10% fetal bovine serum (FBS) with 100 units·mL^−1^ penicillin and 100 μg·mL^−1^ streptomycin (Wisent; Cat. #450‐201‐EL) and maintained at 37 °C incubation with 5% CO_2_. Human MX‐1 breast cancer cells were grown in 1:1 Dulbecco's modified Eagle medium with Ham's F‐12 (DMEM/F12) (Wisent; Cat. #319‐085‐CL) with 5% FBS and 100 units·mL^−1^ penicillin and 100 μg·mL^−1^ streptomycin. All cells were routinely tested for mycoplasma using e‐Myco™ valid Mycoplasma PCR detection kit (iNtRON Biotechnology, Metuchen, NJ, USA; Cat. #25239) according to manufacturer's protocol and authenticated by STR profiling (GenePrint®) at SickKids (Toronto, Ontario); all experiments were performed with mycoplasma‐free cells.

MCF‐7 and MDA‐MB‐231BR cells were transfected with Lipofectamine 2000 (Invitrogen, Waltham, MA, USA, Cat. #11668019) and MDA‐MB‐231 and MX‐1 cells with jetPRIME (Polyplus Transfection, Illkirch, France; Cat. #114–07). Where indicated, cells were stimulated with 10 ng·mL^−1^ of recombinant human EGF (PeproTech, Cranbury, NJ, USA; Cat. #AF‐100‐15) for 10 min, or 100 μm pervanadate for 10 min. Indomethacin (Sigma‐Aldrich; Cat. #17378) was dissolved in DMSO, and applied to cells in serum‐free media at concentrations of 400, 800 and 1000 μm for 24 h prior to stimulation with EGF. Cells were then washed with 5 mL of chilled 1× phosphate‐buffered saline (PBS; pH 7.4) and lysed in 300 μL of phospholipase C (PLC) buffer (10% glycerol, 50 mm Hepes, 150 mm NaCl, 1.5 mm MgCl_2_, 1 mm EGTA, 10 mm NaPP_i_, 100 mm NaF, and 1% Triton X‐100) supplemented with 10 μg·mL^−1^ aprotinin, 10 μg·mL^−1^ leupeptin, 1 mm sodium orthovanadate, and 1 mm phenylmethylsulfonyl fluoride (PMSF). Lysates were centrifuged at 14 000 ***g*** for 12 min at 4 °C, and the supernatant was removed and stored as whole cell lysate (WCL) at −20 °C.

### 2.4 Stable cell line generation

Optimal concentrations of G418 (ThermoFisher Scientific; Cat. #10131035) and puromycin (Sigma; Cat. #P8833 or Wisent; Cat. #400‐160‐EM) for each line were first determined by the kill curve method. Stable cell lines were generated in the MDA‐MB‐231, MDA‐MB‐231BR, and MCF‐7 breast cancer cell lines using two methods: (i) classical system and (ii) sleeping beauty system [19]. For (i), pcDNA3 plasmids encoding either ShcD wild‐type (ShcD^WT^), ShcD mutant (ShcD^PTB*/SH2*^), or pcDNA3‐3×FLAG vector alone were transfected into cells using Lipofectamine 2000, and successful genomic integration and expression were achieved using G418 selection. MDA‐MB‐231 and MDA‐MB‐231BR cells were subsequently maintained in 700 μg·mL^−1^ G418, and MCF‐7 in 400 μg·mL^−1^ G418 diluted in DMEM supplemented with 10% FBS. For (ii), the sleeping beauty pSBbi plasmids expressing FLAG‐tagged ShcD wildtype (ShcD^WT^), ShcD mutant (ShcD^PTB*/SH2*^) or FLAG‐alone were co‐transfected with a transposase encoding vector (pCMV(CAT)T7‐SB100) [22], a gift from Zsuzsanna Izsvak (Addgene; Plasmid #34879) at a ratio of 20:1 (expression vector: transposase). Cells were selected and maintained in 10% FBS DMEM supplemented with 0.5 μg·mL^−1^ puromycin to retain positive selection pressure.

### 2.5 Generation of ShcD‐KO CRISPR pools

hShcD CRISPR‐Cas9 knockout lines were generated using the pSpCas9(BB)‐2A‐Puro (PX459) V2.0 plasmid system, as described in Ran *et al*. [23] and provided as a gift from Feng Zhang (Addgene; Plasmid #62988). Briefly, 20 nucleotide sgRNA guide sequences were designed using the CRISPR design tool in Benchling (https://benchling.com). Selection criteria for sgRNA fragments included “on‐target” and “off‐target” scores >60, location within the exon of interest, and GC content around 50%. The final sequences used to target ShcD/*SHC4* were FW: CACCGTTGGACGAAGGTAGCTCCGG and RV: aaacCCGGAGCTACCTTCGTCCAAC. Selected oligonucleotide primer pairs were incubated with T4 polynucleotide kinase (New England BioLabs; M0201) in T4 DNA polymerase buffer (New England BioLabs; M0203) for 30 min at 37 °C to induce 5′ phosphorylation. Phosphorylated primers were then placed in a 95 °C heat block for 5 min, after which the block was removed from the heat and allowed to cool to 40 °C, and primers were diluted 1:250 in PCR water. Phosphorylated primer pairs were ligated with BbsI‐digested (ThermoFisher Scientific; Cat. #FD1014), alkaline phosphatase‐treated (ThermoFisher Scientific; Cat. #EF0651) PX459 plasmid using T4 DNA ligase (New England BioLabs; Cat. #B0202S) and transformed into DH10b competent cells (New England BioLabs; Cat. #C3019I). Correct insertion of sgRNA oligos was confirmed via Sanger sequencing alignment using Benchling. A human non‐targeting control vector (NT‐Control) was generated in the same manner using sequences of FW: CACCGCACTACCAGAGCTAACTCA and RV: aaacTGAGTTAGCTCTGGTAGTGC [24].

NT‐control and ShcD‐KO pooled CRISPR lines were generated in MX‐1 breast cancer cells. Briefly, cells were transfected with the PX459v2 plasmid containing NT‐control or ShcD‐targeting guide sequences using jetPRIME (Polyplus Transfection; Cat. #114–07) and treated with 2 μg·mL^−1^ of puromycin in complete media for 24–48 h. Knockout efficiency was confirmed via Tracking of Indels by Decomposition (TIDE) analysis [25] of Sanger sequencing on PCR products generated from genomic DNA samples using the primers FW: CTTCGGTTGATCCAGCGGTA and RV: TTCGGAACGAGTCCATCACG, and the loss of protein was confirmed via immunoprecipitation/immunoblotting.

### 2.6 Immunoprecipitation and immunoblotting

Immunoprecipitations (IP) were performed for 2 h while rocking at 4 °C using 400 μL of cell lysate added to a mixture of primary antibody and 10% antimouse IgG‐agarose beads (Sigma; Cat. #A6531) or 20% Protein A Sepharose™ CL‐4B beads (GE Healthcare, Chicago, IL, USA; Cat. #17–0780‐01). Following supernatant removal, beads were washed three times with 800 μL supplemented PLC buffer, and protein complexes were eluted in 2× SDS loading buffer and denatured at 100 °C for 10 min prior to separation via sodium dodecyl sulfate‐polyacrylamide gel electrophoresis (SDS/PAGE). WCLs were prepared for SDS/PAGE by diluting 80 μL in 20 μL of 5× SDS loading buffer (50% glycerol, 300 mm Tris, 10% SDS, and 25% 2‐mercaptoethanol) and boiling at 100 °C for 10 min. For immunoblotting, prepared samples were resolved on 8% or 10% SDS polyacrylamide gels and then transferred to polyvinylidene fluoride (PVDF) membranes (Millipore, Burlington, MA, USA; Cat. #IPVH00010). Membranes were blocked for 30 min in 1× tris‐buffered saline containing Tween 20 (TBST) (0.02 m Tris, 0.15 m NaCl, 0.5% Tween20) with 5% bovine serum albumin (BSA) and incubated with primary antibody at 4 °C. Following an overnight incubation period, membranes were washed three times for 8 min in 1× TBST and incubated with the corresponding secondary antibody for 1 h at room temperature. Membranes were then washed three times for 8 min with 1× TBST. Immunoblot detection was performed using ECL Western blotting substrate (Pierce, Biotechnology, Waltham, MA, USA; Cat. #32106) and imaged using the ChemiDoc™ XRS+ imaging system (Bio‐Rad Laboratories, Hercules, CA, USA).

### 2.7 WST‐1 cell viability assay

Cells were seeded in triplicate or quadruplicate in a 96‐well plate containing 10% FBS DMEM at a cell density of 4000 MDA‐MB‐231 cells/well, 8000 MX‐1 cells/well, and 10,000 MCF‐7 cells/well. Following a 16‐h incubation at 37 °C with 5% CO_2_, 10 μL of WST‐1 colorimetric reagent (Cat. # 05‐015‐944‐001; Roche) was added to each well and incubated for 30 min (*t* = 0). Absorbance values (540 nm and 630 nm) were measured at the indicated time points over 24 h to quantify cell viability per manufacturer's instructions.

### 2.8 Transwell invasion assay

Bottoms of Corning Transwell inserts (8 μm pore diameter; Cat. #CLS3442) were coated with 20 μg·mL^−1^ fibronectin (Sigma; Cat. #F1141‐1MG) and tops were coated with 0.2 mg·mL^−1^ Corning Matrigel (Cat. #354234). 1.5 × 10^5^ cells were seeded in each transwell and placed in 24‐well cell culture plates in DMEM containing 2% FBS in the upper chamber of each well and DMEM containing 10% FBS in the lower chamber. Cells were allowed to invade into the Matrigel for 20 h (MDA‐MB‐231), 24 h (MX‐1), or 40 h (MCF‐7) at 37 °C, following which the transwell membranes containing invaded cells were removed. The membranes were then fixed and stained with 1% crystal violet in a 20% methanol solution. Following three successive washes with 1× PBS, the crystal violet was eluted from the membranes in 10% acetic acid. Finally, the absorbance (595 nm) of eluants was measured to quantify invasion.

### 2.9 Adhesion assay

The wells of 12‐well plates were coated with 5 μg·mL^−1^ of fibronectin (Sigma; Cat. # F1141) and seeded with 1.5 × 10^5^ MDA‐MB‐231 or MX‐1 cells in duplicate. After 2 h, the cells were washed four times with 1× PBS, then fixed and stained with 1% crystal violet (Fisher, Waltham, MA, USA; Cat. #C581‐100) diluted in 20% methanol. Following another series of PBS washes, crystal violet‐stained cells were eluted in 10% acetic acid for 30 min. Eluants were measured at 595 nm and blanked to unseeded stained wells. Alternatively, images were taken through a 10× objective lens using a Leica DMIRE2 epifluorescence microscope. Images (10 random fields of view per duplicate) were processed and analyzed using the fiji software (National Institutes of Health, Bethesda, MD, USA).

### 2.10 Generation of cerebral organoids

The iPSC line used in our study was previously generated from one healthy adult control subject with informed consent (Massachusetts General Hospital Department of Psychiatry) [26]. Cerebral organoids were generated from iPSCs using the STEMdiff™ Cerebral Organoid Kit (Stem Cell Technologies, Vancouver, Canada; Cat. #08570), according to the manufacturer's protocol and maintained until they reached day 55–65 of maturation; then similarly sized organoids were selected and used for tumor spheroid–cerebral organoid assembloid generation.

### 2.11 Generation of tumor spheroids

Tumor spheroids were generated from MDA‐MB‐231BR cells stably expressing FLAG‐alone, ShcD,^WT^ or ShcD^P*/S*^. First, cells were transduced with lentiviral particles expressing EGFP using pWPXLd‐EGFP, a gift from Didier Trono (Addgene; Plasmid #12258). Next, 10,000 cells·well^−1^ were seeded into a 96‐well round‐bottom ultra‐low attachment plate (*n* = 15 per cell line) and left to form spheroids over a 5‐day period. Consistently sized spheroids were selected for assembloid generation.

### 2.12 Generation of assembloids

Assembloids were generated by adding one cerebral organoid on top of three to four tumor spheroids in a 1.5‐mL Eppendorf tube filled with 1 mL Maturation medium (Stem Cell Technologies; Cat. #08571). At least three assembloids were generated per cell line. Assembloids were grown in Eppendorf tubes for 3–4 days to assure attachment of tumor spheroids onto cerebral organoids, then moved to 24‐well ultra‐low attachment plates for another 10 days before immunostaining analysis.

### 2.13 Assembloid co‐culture immunofluorescence and image analysis

Cerebral organoid‐spheroid assembloid cocultures were transferred to 50‐mL conical tubes, washed three times with 1× PBS, then fixed in 4% paraformaldehyde overnight at 4 °C. Specimens were washed three times with 1× PBS, followed by overnight incubation at 4 °C in a 30% sucrose solution. The next day, organoids were embedded in a 7.5% gelatin/10% sucrose solution and snap‐frozen using a dry‐ice/ethanol bath. 30‐μm‐thick sections were sliced using a Leica CM1860 cryostat, captured on Vectabond‐coated glass slides (Vector Laboratories, Newark, CA, USA, SP‐1800‐7), air‐dried, and then maintained at −80 °C until histological processing. For immunostaining, sections were washed three times with 1× PBS for 10 min each, blocked with 1× PBS containing 10% goat serum for 30 min, followed by overnight incubation at 4 °C with the primary antibody solution containing mouse anti‐TUBB3 (clone‐Tuj1) (Biolegend, San Diego, CA, USA; Cat. #801213) and chicken anti‐GFP (Invitrogen; Cat. #A10262). The next day, sections were washed three times in 1× PBS, followed by incubation for 2 h at room temperature in Alexa‐488‐ or Alexa‐647‐conjugated antisera (ThermoFisher Scientific, Waltham, MA, USA, Cat. #A11039 and #A21235, respectively) solution. After incubation, sections were briefly washed with 1× PBS, counterstained with 4′,6‐diamidino‐2‐phenylindole (DAPI), and immediately cover‐slipped with ProLong AntiFade mounting medium (Molecular Probes, Waltham, MA, USA; Cat. #P36961).

Up to 15 sections were collected for each group from three organoids, which were then imaged and analyzed as reported previously [15]. Overlapping grayscale images were collected using a 4× or 20× objective and a Nikon Eclipse Ti2‐E inverted microscope (Nikon Instruments, NY, USA) equipped with a motorized stage and image stitching capability. Image deconvolution and analysis were conducted using NIS‐Elements Viewer software (Nikon Instruments) and imagej/fiji. Briefly, breast cancer cell invasion into the organoids was quantified using imagej software by first overlaying digital images acquired from GFP (GFP‐expressing MDA‐MB‐231BR cells) and Cy5 channels (Tuj1‐positive organoid) and employing color thresholding in order to calculate the dispersion of cancer cells within the organoid. The organoid surface was then approximated based on the area measurement of Tuj1‐positive cells. To measure the area covered by invading breast cancer cells, hue settings were arranged to select only co‐localized areas within the total body of the organoid, and pixels for each image set were recorded. The same background/foreground cut‐off threshold was used for all conditions. Finally, percent colocalized areas were calculated using the formula: % Co‐localization = [Area_hue(28–48)_*100/Area_hue(0–225)_].

### 2.14 Affinity‐purification mass spectrometry (AP‐MS) sample preparation and data acquisition

Five 150 mm plates of MDA‐MB‐231 cells expressing ShcD^WT^‐FLAG, ShcD^PTB*/SH2*‐^FLAG, or FLAG‐alone were serum‐starved for 16 h and then stimulated with 100 μm pervanadate for 10 min at 37 °C and harvested for each replicate. The experiment was performed in triplicate. Cell pellets were flash‐frozen. FLAG‐affinity purification and tryptic digest were performed as a service at the Network Biology Collaborative Centre (NBCC) (Toronto, Ontario) using their standard protocols (https://nbcc.lunenfeld.ca/proteomics.html#fourth-section).

One‐third of the total sample was diluted to 20 μL with 5% formic acid for loading onto EvoTip Pure tips for each injection. Tips were loaded following the manufacturer's specifications and then preserved in 0.1% formic acid in water while waiting for analysis. Each sample was injected twice for DDA and DIA on the MS. Samples were analyzed by an EvoSep One liquid chromatography system using the 60 samples per day (60SPD) standard gradient. Mobile phases were also the EvoSep standard consisting of 0.1% formic acid in water and 0.1% formic acid in acetonitrile. Separation was performed on an EvoSep EV1109 Performance column (8 cm × 150 μm, 1.5 μm C18 packing). The column was coupled to a Bruker timsTOF Pro 2 via a 20 μm silica emitter (Bruker Captive Spray ZDV Sprayer 20) and held at 40 °C in a Bruker Column Toaster column oven.

MS data were first collected using data‐dependent Parallel Accumulation Serial Fragmentation (PASEF) acquisition on the timsTOF Pro 2 (DDA). The Captive Spray source was set to a capillary voltage of 1600 V, a 3.0 L·min^−1^ dry gas flow rate, and a temperature of 180 °C. MS/MS triggering was performed using a 4‐frame PASEF cycle with a cycle overlap of 1 and a total cycle time of 0.53 s over an ion mobility range of 0.85 to 1.3 1/K_0_. Active exclusion was enabled with a 0.4 min release delay, and precursor repetitions were enabled with a minimum target intensity of 17500 arbitrary units (a.u.) and an intensity threshold of 1750 a.u. and/or where MS precursor intensity was four times greater than initially recorded. Isolation widths were as per the default settings, with a width of 2 m/z below 700 m/z isolation and a width of 3 m/z above 800 m/z isolation mass. Singly charged precursor ions were excluded by a polygonal filter via timsControl 4 (Bruker). Data independent acquisition (DIA) was performed on a separate injection of the sample with the same separation conditions but with diaPASEF MS acquisition settings. The diaPASEF was set up with 20 windows, each 50 m/z in width, running from 400 m/z to 1350 m/z with a 1 m/z overlap between windows across the 0.85–1.3 inverse mobility range. The total cycle time was 1.05 s. Both DDA and DIA collision energies were selected based on the precursor mobility ranging from 29.56 eV at inverse mobility 0.85–58.09 eV at inverse mobility 1.3.

### 2.15 AP‐MS data search

Mass spectrometry data were stored, searched, and analyzed using the ProHits 7.0 laboratory information management system platform [27] with an integrated FragPipe 19.0 and DIA‐NN 1.8.2 installation [28]. DDA files were first searched using MSFragger directly from the Bruker raw .d files. Files were searched against a UniProt human proteome, with additional contaminant proteins added as well as sequences from common fusion proteins and epitope tags. The sequence database consisted of forward and reversed sequences. Default MSFragger parameters were used throughout, except the upper and lower precursor m/z tolerances were set to 40 m/z, and the fragment m/z tolerance was also set to 40 m/z. Validation was performed using Percolator [29] and MS Booster [30] at their default settings. The DDA search results were used to create a spectral library. The diaPASEF data were then searched against that library using DIA‐NN 1.8.2 [31]. Default search parameters were used, including match between runs, except the quantitation strategy was “Robust LC” and protein inference was disabled. All non‐human protein contaminants were manually removed from the output.

### 2.16 Protein–protein interaction scoring and analysis

Starting from the output of DIA‐NN, any missing values were added as 90% of the lowest reported value for all preys in the replicate based on the methodology of Teo *et al*. [32]. Interactions for each bait were evaluated by comparison of mean intensity for each prey relative to FLAG‐alone control, and statistical significance was determined by multiple t‐tests in Graphpad Prism with a *P* < 0.05 cutoff for significance (Table S1). Non‐specific interactors were removed by comparison with the Crapome database using the “Data filtering” tool at http://www.proteomics.fi/. During our data analysis, we noticed that some established and predicted ShcD interactors fell just outside our statistical cutoff, such as MEMO1 [33]. Therefore, we expanded our initial dataset to include MEMO1 as well as GAB1 and PRKCD, close paralogs of identified interactors GAB2 and PRKCB, respectively.

The significant interactions identified were compared with the literature‐reported interactors for ShcD/SHC4 from BioGrid [34], BioPlex 3.0 [35] and TissueNet v3 [36]. The bait‐bait comparison plot was generated using Prohits Viz [37] (https://prohits‐viz.org) based on mean DIA‐NN intensities, and the subset of interactors with at least 2‐fold enrichment for ShcD^WT^ was subjected to gProfiler [38] analysis (https://biit.cs.ut.ee/gprofiler), followed by manual curation of the results. The complete gProfiler results are provided in Table S2. WikiPathways [39] enrichment of the network consisting of all proteins interacting with ShcD^WT^ was performed using Metascape [40] (https://metascapemetascape.org). EGFR signaling complex components in Fig. 3 were identified based on Chen *et al*. [41] and Wang *et al*. [42].

### 2.17 Gene expression analyses

To visualize ShcD mRNA distribution patterns across a panel of 33 different cancer types, RNA‐Seq hg38 harmonized TCGA datasets (v27.0; Oct 29, 2020) were accessed and retrieved from NCI Genomic Data Commons (GDC) using the *TCGABiolinks* R package as raw HT‐Seq counts. Counts for all tumors were normalized using the TMM (trimmed mean of M‐values) function from the *edgeR* package, transformed using the voom function from the *limma* R package, and visualized as violin plots generated with the *ggplot2* R package.

*SHC4* over‐expression in breast cancer subtypes was analyzed using the above transformed counts for the BRCA dataset tumors, and the same process was also performed for BRCA normal sample counts, resulting in two groups, normal (NT; *n* = 113) and tumor (TP; *n* = 1102). Next, average expression was calculated for normal samples. This served as the baseline for fold change (FC) calculations. For each tumor sample from a patient with a PAM50 subtype of Basal_like, Luminal_A, Luminal_B, or HER2 (*n* = 1050), FC was calculated as follows: logFC = log2(tumor)/log2(normal), which is the same as log2(tumor‐normal). Values indicate the percentage and number of patients overexpressing *SHC4* using a twofold minimum cutoff. Cell line gene expression data (dataset E‐MTAB‐2770) was retrieved from ArrayExpress [43].

### 2.18 Densitometry and statistics

To determine relative immunoblot band intensities, membranes were imaged and quantified using the ImageLab analysis software (versions 2.0–5.2, Bio‐Rad) or fiji software (version 2.9.0, https://imagej.net/software/fiji/) [44]. All statistical calculations were performed and plotted in GraphPad Prism (versions 7–9). For normalized data with matched controls (minimum *n* = 3), two experimental groups were compared by one‐sample t‐test when data passed the Shapiro–Wilk normality test or by Wilcoxon test when data failed. Comparisons of three or more experimental groups were analyzed by Kruskal–Wallis test with Dunn's multiple comparison test. For viability assays, each experimental group was compared to its control by repeated‐measures ANOVA. In all cases, the threshold of significance was set to *P* < 0.05, and error bars represented the standard deviation (SD).

## 3 Results

### 3.1 ShcD/SHC4 mRNA expression is upregulated in human breast cancers

**Fig. 1:**
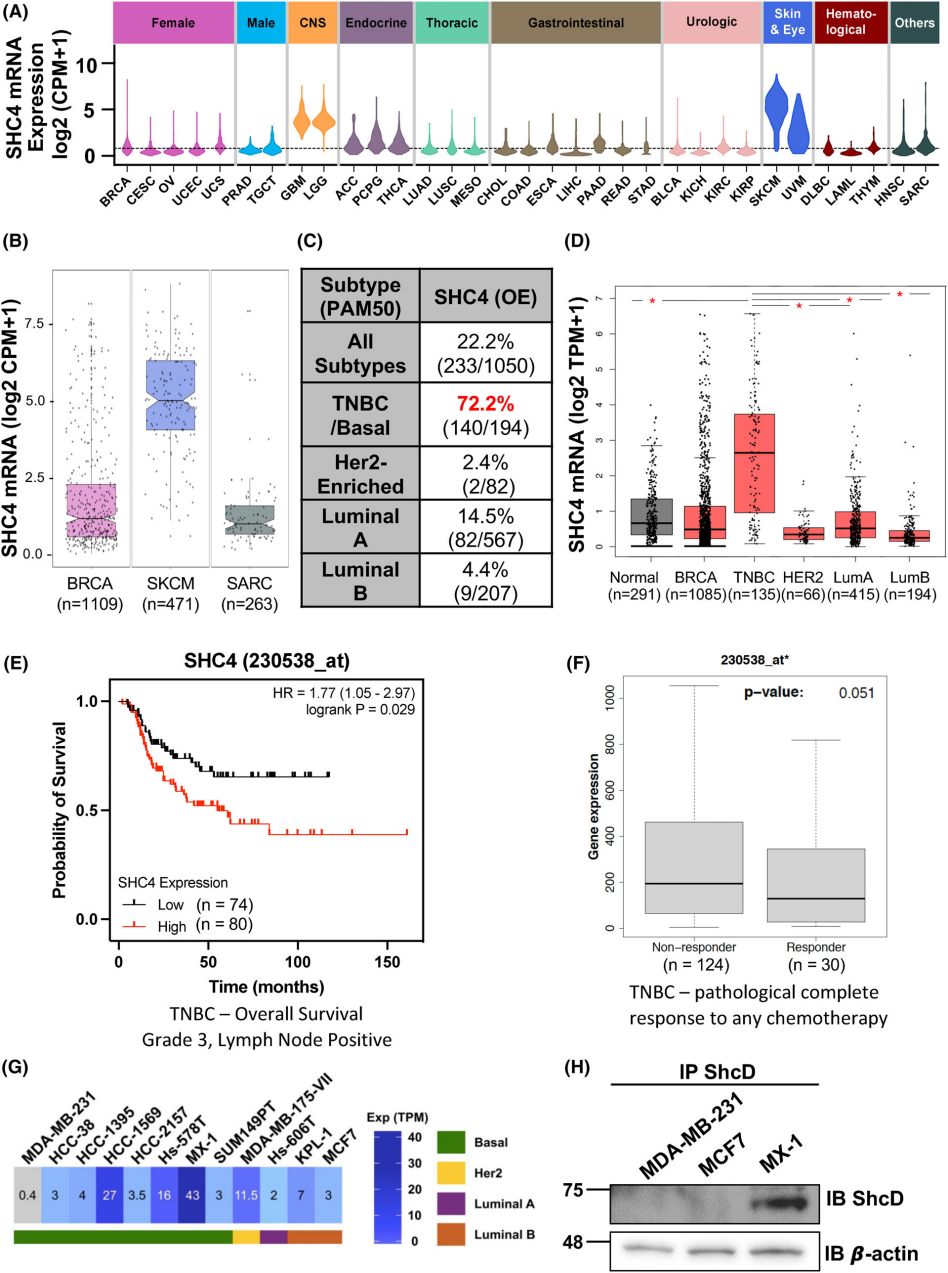
Bioinformatic analyses of ShcD expression show upregulation in human breast cancer. (A) Violin plots showing the distribution of ShcD (*SHC4*) mRNA expression across a panel of 33 cancer types from The Cancer Genome Atlas (TCGA). Dotted line represents the median expression across all 33 cancers including breast invasive carcinoma (BRCA, *n* = 1109), cervical squamous cell carcinoma and endocervical adenocarcinoma (CESC, *n* = 306), ovarian cancer (OV, *n* = 379), uterine corpus endometrial carcinoma (UCEC, *n* = 552), uterine carcinosarcoma (UCS, *n* = 56), prostate adenocarcinoma (PRAD, *n* = 499), tenosynovial giant cell tumor (TGCT, *n* = 156), glioblastoma multiforme (GBM, *n* = 169), brain lower grade glioma (LGG, *n* = 529), adrenocortical carcinoma (ACC, *n* = 79), pheochromocytoma and paraganglioma (PCPG, *n* = 183), thyroid carcinoma (THCA, *n* = 510), lung adenocarcinoma (LUAD, *n* = 535), lung squamous cell carcinoma (LUSC, *n* = 502), mesothelioma (MESO, *n* = 86), cholangiocarcinoma (CHOL, *n* = 36), colon adenocarcinoma (COAD, *n* = 480), esophageal carcinoma (ESCA, *n* = 162), liver hepatocellular carcinoma (LIHC, *n* = 374), pancreatic adenocarcinoma (PAAD, *n* = 178), rectum adenocarcinoma (READ, *n* = 167), stomach adenoma carcinoma (STAD, *n* = 375), bladder urothelial carcinoma (BLCA, *n* = 414), kidney chromophobe renal cell carcinoma (KICH, *n* = 65), kidney renal clear cell carcinoma (KIRC, *n* = 539), kidney renal papillary cell carcinoma (KIRP, *n* = 289), skin cutaneous melanoma (SKCM, *n* = 471), uveal melanoma (UVM, *n* = 80), lymphoid neoplasm diffuse large B‐cell lymphoma (DLBC, *n* = 48), acute myeloid leukemia (LAML, *n* = 151), thymoma (THYM, *n* = 119), head and neck squamous cell carcinoma (HNSC, *n* = 502), and sarcoma (SARC, *n* = 263). (B) Box plot of *SHC4* expression in BRCA, SKCM, and SARC datasets from A showing individual patient values. (C) Percent of patients with *SHC4* overexpression (OE) of the 1050 patients with a known breast cancer subtype as indicated based on the BRCA dataset in A. (D) GEPIA2 analysis of *SHC4* expression in TCGA‐BRCA normal/control samples, all TCGA‐BRCA samples, and within individual BRCA subtypes. Data and analysis from GEPIA2 (http://gepia2.cancer‐pku.cn). Accessed January 2023. (E) High ShcD/*SHC4* levels are associated with low survival rates in triple‐negative breast cancer (TNBC)/Basal Grade 3 lymph‐node positive breast cancer patients. Data and analysis from kmplot.com. Accessed May 2023. (F) Increased ShcD/*SHC4* levels are observed in TNBC patients who do not respond to chemotherapy compared to those whose cancer is responsive to chemotherapy. Data and analysis from ROCplot.org. Accessed May 2023. (G) The EMBL‐EBI gene expression atlas (dataset E‐MTAB‐2770) was used to profile *SHC4* RNA expression levels in human breast cancer cell lines. Accessed February 2021. (H) Immunoprecipitation (IP) and immunoblot (IB) analysis of endogenous ShcD protein expression in MDA‐MB‐231, MCF7, and MX‐1 human breast cancer cells.

Shc adaptors are associated with multiple cancers [13]; however, the role of ShcD is less well characterized. We therefore conducted a broad survey of ShcD mRNA (*SHC4*) expression levels in TCGA RNA‐seq datasets across a representative panel of 33 human cancer types, where we detected its upregulation in melanomas (SKCM, UVM) and gliomas (GBM, LGG), consistent with previous reports [14, 15, 16, 17] (Fig. 1A). Upregulation of *SHC4* was also observed in both breast (BRCA) and sarcoma (SARC) cancers, though there was a large degree of variability in transcript levels, suggesting there could be increased expression within specific tumor subtypes. Indeed, when we looked more closely at the three cancers with the highest expression of *SHC4* (BRCA, SKCM and SARC), many BRCA samples had expression levels >2.5, as was seen with SKCM, whereas few SARC samples exceeded that threshold (Fig. 1B). Given these profiles and the known role of ShcA in breast cancer [45, 46], we chose to further investigate ShcD in BRCA.

Given the heterogeneity within breast cancer subtypes, we compared *SHC4* between normal tissue and all breast cancers, as well as within subtypes. When we examined *SHC4* transcripts within all breast cancer subtypes in the TCGA‐BRCA dataset using RNA‐seq PAM50 subtype signatures, we found *SHC4* mRNA was upregulated at least 2‐fold compared to normal tissue in 22% of samples; however, this proportion was dramatically increased to >70% of TNBC/Basal subtype breast tumor cases, with much lower rates in HER2‐enriched and luminal A/B subtypes (Fig. 1C). We then turned to the GEPIA2 portal [47] and, using their TCGA‐BRCA analysis platform, similarly observed significantly increased *SHC4* levels in TNBC/Basal‐like samples compared to normal breast samples as well as to each of the other subtypes (Fig. 1D). An increased level of *SHC4* in TNBC versus non‐TNBC samples was reproducible in both the METABRIC [48] (Fig. S1A) and SCAN‐B [49] datasets (Fig. S1B) in MammOnc‐DB [50], and we confirmed this finding more broadly using the Gent2 database, which contains >2000 patients [51] (Fig. S1C).

Given overall outcomes can differ between subtypes, we next checked kmplot.com [52] for any relationship between *SHC4* levels and metastasis using TNBC Grade 3 lymph‐node positive patients as a surrogate marker of metastatic progression [53] and found that high levels of *SHC4* are correlated with decreased overall survival in this cohort (Fig. 1E), with a smaller effect persisting across all breast cancer subtypes (Fig. S1D). Additionally, analysis of ROCplot.org [54] data shows that TNBC patients who do not respond to chemotherapy treatment have increased expression of *SHC4* compared to responding patients (Fig. 1F). Altogether these results suggest that ShcD/*SHC4* is upregulated in TNBC and that high ShcD/*SHC4* levels correlate with poor patient outcomes.

Based on these results, we next queried ShcD/*SHC4* mRNA levels in breast cancer cell lines of public datasets [55] (Fig. 1G; Fig. S1E). There was a range of expression, with the highest levels found in MX‐1 TNBC cells. In line with these observations, we compared ShcD protein levels in several breast cancer cell lines (Fig. 1H) and found they generally correlated. Indeed, there were low levels of endogenous ShcD present in the MDA‐MB‐231 TNBC cell line and in the luminal MCF‐7 line, with higher levels in the MX‐1 line. The existence of TNBC lines with high (MX‐1) and low (MDA‐MB‐231) levels of ShcD provides an opportunity to investigate whether manipulation of ShcD expression—by overexpression of ShcD in MDA‐MB‐231 s or knockdown of ShcD in MX‐1 cells—can influence breast cancer cell invasion, as we reported in the context of gliomas [15].

### 3.2 ShcD overexpression increases breast cancer cell invasion and adhesion through PTB/SH2 domain binding contributions

**Fig. 2:**
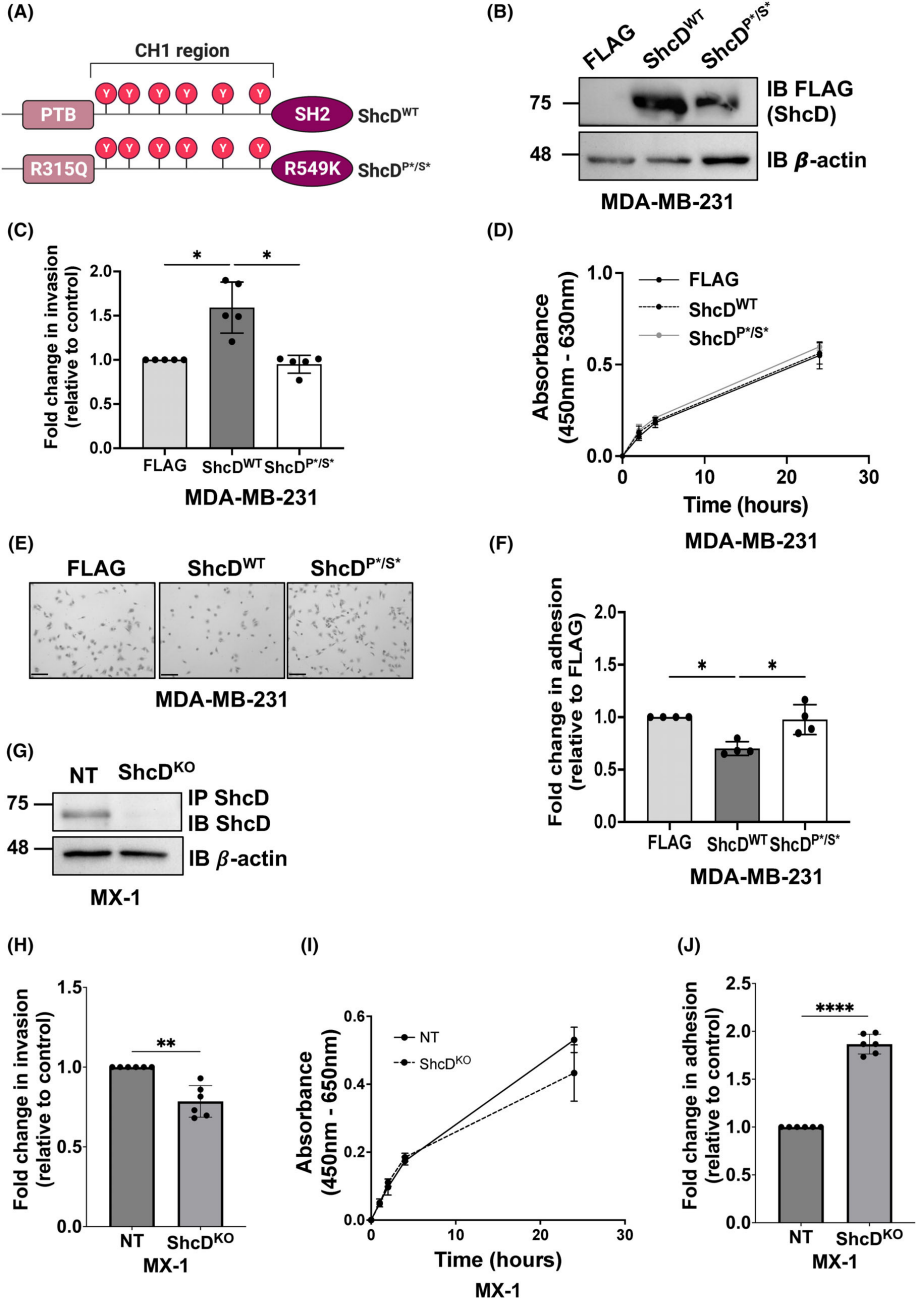
ShcD influences invasive and adhesive properties of breast cancer cells through phosphotyrosine binding domain contributions. (A) Schematic depiction of ShcD wildtype (ShcD^WT^) and mutant protein architecture.^P*/S*^s point mutations in the phosphotyrosine binding (PTB) (R315Q) and Src‐homology 2 (SH2) (R549K) domains that disable binding function. (B) Immunoblot (IB) characterizing expression of FLAG‐tagged ShcD^WT^ and ShcD^P*/S*^ in stable MDA‐MB‐231 cells. (C) Stable MDA‐MB‐231 cellswere serum‐starvedd for 24 h then seeded into Matrigel‐coated Boyden chambers and allowed to invade for 20 h (*n* = 5). (D) Cell viability was measured in parallel to ensure consistent growth rates (*n* = 5). (E) Representative images from cells seeded on fibronectin and left to adhere for 2 h (*n* = 4). Scale bar = 170 μm. (F) Quantification of E (*n* = 4). (G) ShcD CRISPR knockout (KO) and non‐targeting (NT) control MX‐1 triple‐negative breast cancer (TNBC) cells were generated and verified for expression by immunoblot. (H) MX‐1 cellswere serum‐starved for 24 h and seeded into Matrigel‐coated Boyden chambers and allowed to invade for 24 h (*n* = 6). (I) Cell viability was measured in parallel to ensure consistent growth rates (*n* = 6). (J) MX‐1 cells were seeded on fibronectin and left to adhere for 2 h (*n* = 6). The data represent a minimum of four independent experiments; *P < 0.05; **P < 0.01; ****P < 0.001. Error bars represent standard deviation (SD). (C, F) *P*‐values represent significance levels from Kruskal‐Wallis ANOVA followed by Dunn's multiple comparison test. (D) *P*‐values represent significance levels from repeated measures two‐way ANOVA. Data shown as mean ± SD.

ShcD signaling is dependent on its phosphotyrosine binding PTB and SH2 domains; therefore, we took advantage of our existing constructs to generate MDA‐MB‐231 cell lines overexpressing either wild‐type (WT) or the PTB and SH2 signaling‐deficient mutant version of ShcD (P*/S*) (Fig. 2A). Using the sleeping beauty system [19], we produced stable cell lines expressing WT FLAG‐tagged ShcD (ShcD^WT^), mutant ShcD (ShcD^P*/S*^) or FLAG‐alone and verified similar levels of ShcD‐FLAG expression (Fig. 2B). Next, stable cells were assayed for invasion in Matrigel‐coated Boyden transwells toward a gradient of serum over a 20‐h period. Overexpression of ShcD^WT^ enhanced cell invasion around 1.5‐fold; however, cells expressing the ShcD^P*/S*^ mutant displayed similar levels of invasion as FLAG‐alone control cells (Fig. 2C). We verified that this increase in invasion in ShcD^WT^ cells was not due to an enhanced growth rate (Fig. 2D). Moreover, we also confirmed that ShcD^WT^ overexpression had a similar effect on invasion in a non‐TNBC cell line using the weakly invasive MCF‐7 cell line [56] (Fig. S2A–C). Considering that changes in matrix adhesion are another key feature of metastatic progression, we also assessed whether ShcD overexpression had any influence on cell adhesion. MDA‐MB‐231 stable cell lines were seeded on fibronectin‐coated plates and allowed to adhere over a 2‐h period. ^WT^ShcD‐expressing cells had reduced adhesion to fibronectin compared to ShcD^P*/S*^ and FLAG‐alone‐expressing cells, which displayed similar levels of adherence (Fig. 2E,F). Together, these results demonstrate that heightened levels of ShcD in breast cancer enhance cell invasion and that these invasive capabilities require the ability of ShcD to engage upstream RTKs via its PTB and SH2 binding domains.

Lastly, we took advantage of high endogenous ShcD expression in MX‐1 TNBC cells and used CRISPR/Cas9 gene editing to generate stable pooled ShcD knockout (KO) and nontargeting (NT) control cell lines (Fig. 2G; Fig. S3A,B). We similarly assayed invasion, viability, and adhesion in these lines (Fig. 2H–J) and found that ShcD KO decreases invasion and increases adhesion, in opposition to our findings in the overexpression model.

### 3.3 Defining a ShcD breast cancer‐specific EGFR‐centric signaling network

**Fig. 3:**
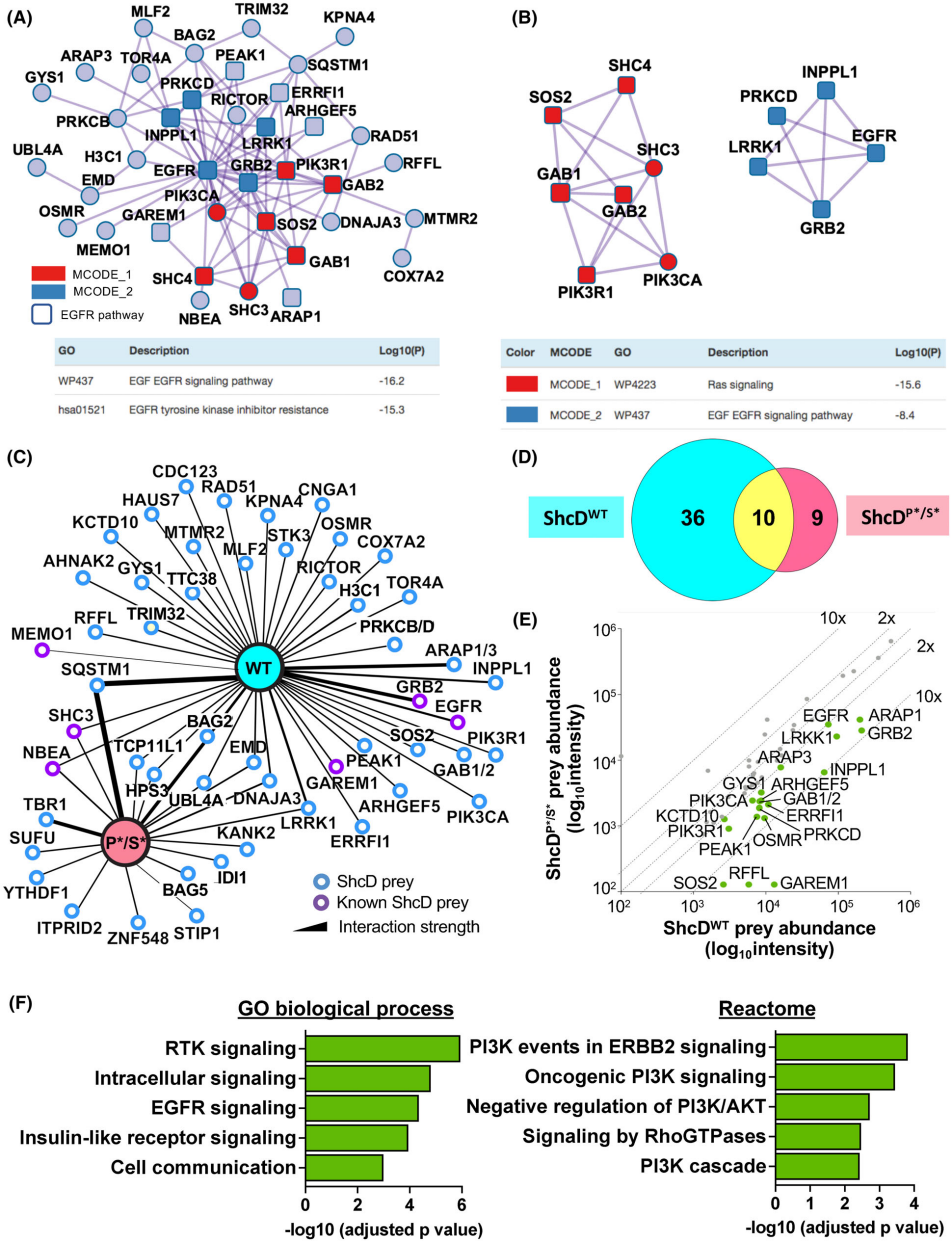
The ShcD breast cancer interactome. (A) Metascape‐generated network of wildtype ShcD (ShcD^WT^) preys identified by affinity‐purification mass spectrometry (AP‐MS) in MDA‐MB‐231 cells, with epidermal growth factor receptor (EGFR) signaling partners shown in squares and enriched subnetworks in the indicated colors. (B) Top MCode enriched subnetworks and their significantly enriched WikiPathways from Metascape analysis. (C) Network of ShcD^WT^ (WT) and ShcD^P*/S*^ (P*/S*) preys identified in MDA‐MB‐231 cells. (D) Comparison (Euler diagram) of overlap between ShcD^WT^ and ShcD^P*/S*^ preys. (E) Differential prey abundance between ShcD^WT^ and ShcD^P*/S*^ baits. Preys with greater than two‐fold higher ShcD^WT^ abundance are shown in green. (F) Selected highly enriched GO Biological Processes and Reactome terms from gProfileR pathway analysis of the green preys within the ShcD^WT^ interactome shown in E (*n* = 3).

Next, to identify molecular mechanisms governing ShcD function in breast cancer cells, we used a proteomic approach to screen for putative ShcD binding partners in MDA‐MB‐231 cells. Lysates from ShcD^WT^ and FLAG‐alone control cells were subjected to affinity‐purification mass‐spectrometry (AP‐MS) to detect significant interactions, which were analyzed with Metascape to generate a densely connected network (Fig. 3A) containing several well‐characterized ShcD binding partners including EGFR, Grb2 and Sos. Indeed, the network is highly enriched in EGFR signaling partners (squares), which comprise around one third of total interactors, in line with the top pathways of “EGF EGFR signaling” and “EGFR tyrosine kinase inhibitor resistance.” Metascape M‐Code analysis also identified two highly enriched subnetworks (Fig. 3B) composed of “Ras signaling” and “EGF EGFR signaling” components.

To determine which binding partners might be important for the invasive functions of ShcD, we also performed AP‐MS with our ShcD^P*/S*^ stable line. A comparison of binding partners shared between ShcD^WT^ and ShcD^P*/S*^ (Fig. 3C,D) shows a loss of interactions with EGFR and other signaling components such as Grb2, as anticipated, although a small number of interactors remain associated with ShcD^P*/S*^. To more accurately compare relative interaction strength between wildtype and mutant ShcD, we performed a bait:bait comparison that revealed that over 40% of interactors were enriched specifically with ShcD^WT^ as compared to ShcD^P*/S*^ (Fig. 3E), a group that includes several regulators of PI3K/Akt signaling that were also identified as key hubs by Metascape (Fig. 3A). The role of these ShcD^WT^‐enriched interactors in EGFR and Akt signaling is likewise reflected within the terms produced by functional enrichment analysis using gProfiler (Fig. 3F).

### 3.4 ShcD interacts with upstream regulators of PI3K and suppresses Akt phosphorylation downstream of EGFR

**Fig. 4:**
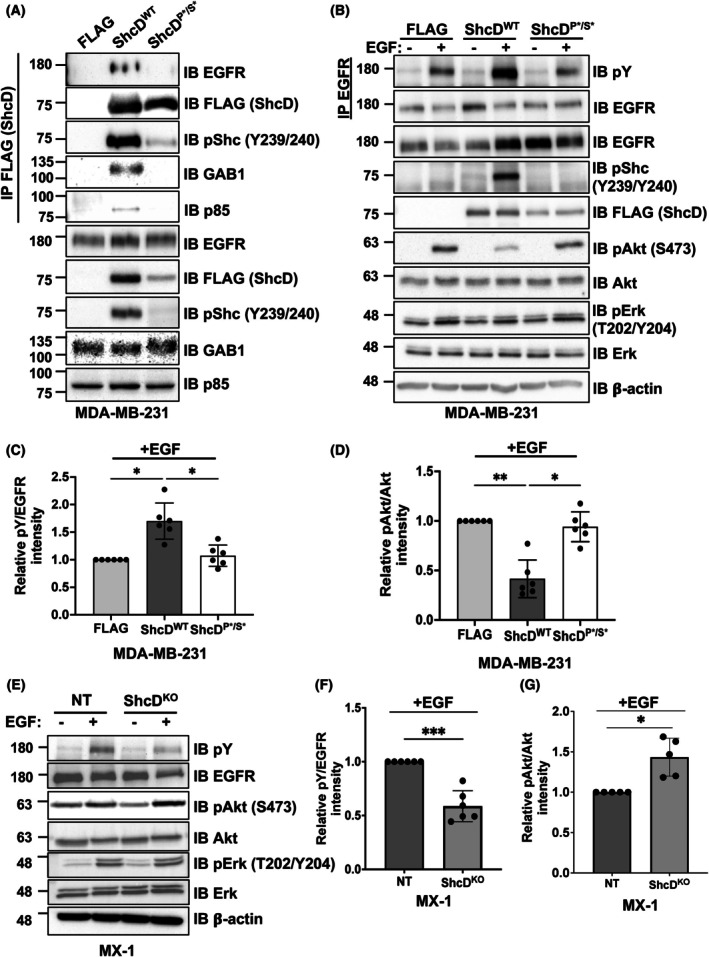
ShcD interacts with components of the PI3K/Akt pathways in breast cancer cells, heightens EGFR tyrosine phosphorylation, and represses downstream Akt phosphorylation. (A) FLAG immunoprecipitants and whole cell lysate from pervanadate‐treated (100 μm for 10 min) MDA‐MB‐231 cells stably expressing FLAG‐alone, FLAG‐tagged wildtype ShcD (ShcD^WT^) or FLAG‐tagged mutant ShcD (ShcD^P*/S*^) were probed with antibodies targeting epidermal growth factor receptor (EGFR), ShcD, pShc, GAB1, and p85 (*n* = 4). (B) Stable MDA‐MB‐231 cells expressing FLAG‐alone, ShcD,^WT^ or ShcD^P*/S*^ were stimulated with epidermal growth factor (EGF) (10 ng·mL^−1^; 10 min) and profiled for changes in EGFR, phosphotyrosine (pY), ShcD, pShcD, Erk, pErk, Akt, and pAkt by immunoprecipitation (IP) and immunoblotting (IB) (*n* = 6). (C) Densitometric analysis of EGFR tyrosine phosphorylation levels in ^WT^ShcD‐expressing cells compared to control and mutant‐expressing cells in C (*n* = 6). (D) Densitometric analysis of Akt phosphorylation levels in ^WT^ShcD‐expressing cells compared to control and mutant‐expressing cells in C (*n* = 6). (E) MX‐1 non‐targeting (NT) and ShcD knockout (KO) cells were stimulated with EGF (10 ng·mL^−1^; 10 min) and profiled for changes in levels of EGFR and pY by immunoblotting (*n* = 5). (F) Densitometric analysis of EGFR tyrosine phosphorylation levels (pY) in NT cells compared to ShcD KO cells in E (*n* = 6). (G) Densitometric analysis of Akt phosphorylation levels in NT cells compared to ShcD KO cells in E (*n* = 5). The data represent a minimum of four independent experiments; *P < 0.05; **P < 0.01; ***P < 0.001. (C, D) *P*‐values represent significance levels from Kruskal‐Wallis ANOVA followed by Dunn's multiple comparison test. (F, G) *P*‐values represent significance levels from one‐sample *t*‐test. Data shown as mean ± SD.

Since dysregulated EGFR/PI3K/Akt signaling contributes to breast cancer development and progression [57], we sought to confirm interactions between ShcD and this group of candidate binding partners. Using our stable MDA‐MB‐231 cell lines treated with pervanadate to preserve tyrosine phosphorylation, we found that endogenous EGFR, GAB1, and PI3K‐subunit p85 (PIK3R1) co‐immunoprecipitated with ShcD^WT^ but not with the mutant ShcD^P*/S*^, which is only weakly phosphorylated on its central tyrosine residues (Fig. 4A). We then compared downstream signaling responses following EGF stimulation in ShcD^WT^, ShcD,^P*/S*^ and FLAG‐alone cells (Fig. 4B). In line with our previous findings, overexpression of ShcD induced RTK hyperphosphorylation, with ShcD^WT^ cells showing an approximate twofold increase in EGFR tyrosine phosphorylation following EGF treatment compared to ShcD^P*/S*^ or FLAG‐alone cells (Fig. 4C). Intriguingly, this increase in EGFR phosphorylation was associated with an approximate 2‐fold reduction in downstream Akt phosphorylation (Fig. 4D). While these findings were unexpected as EGFR hyperphosphorylation is typically associated with the activation of downstream effectors, they are consistent with observations from our proteomic analysis wherein negative regulation of the PI3K/Akt network is a highly enriched pathway (Fig. 3F). We also assessed Erk phosphorylation after EGF stimulation but detected no differences across groups, despite prominent upstream phosphorylation of EGFR and ShcD in ShcD^WT^ cells (Fig. 4B). We have previously reported that ShcD signaling suppresses Erk phosphorylation [58]; however, these results suggest this effect may be context‐dependent.

By contrast, in the ShcD KO setting of MX‐1 cells, EGF‐induced EGFR phosphorylation was significantly reduced in ShcD KO cells compared to NT controls (Fig. 4E,F). Moreover, in opposition to our overexpression model, Akt phosphorylation was increased in ShcD KO cells compared to NT cells (Fig. 4G) with no effect seen on Erk phosphorylation. Altogether, these findings correlate increased ShcD expression with the activation of EGFR signaling and suppression of Akt in breast cancer cells.

### 3.5 MDA‐MB‐231 brain metastatic spheroids expressing ShcD show an enhanced invasive propensity

**Fig. 5:**
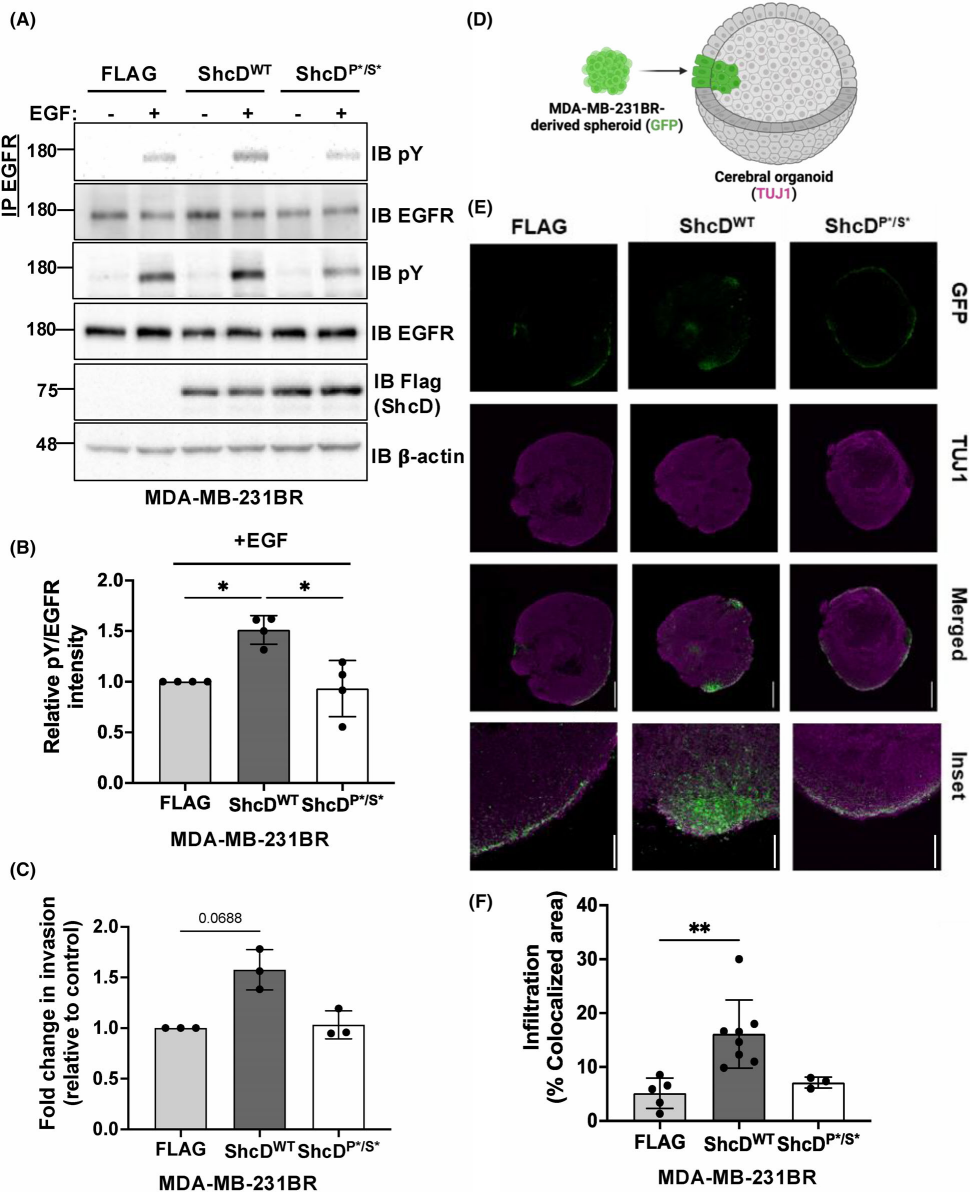
Malignant brain‐targeting MDA‐MB‐231BR breast cancer cells expressing ShcD show deregulated EGFR signaling and heightened infiltration into cerebral organoids. (A) Stable MDA‐MB‐231BR cells expressing FLAG‐tagged wildtype ShcD (ShcD^WT^), mutant ShcD (ShcD^P*/S*^) or FLAG‐alone were stimulated with epidermal growth factor (EGF) (10 ng·mL^−1^; 10 min) and probed for indicated proteins by immunoprecipitation (IP) and immunoblotting (IB) (*n* = 4). (B) Densitometric analysis of epidermal growth factor receptor (EGFR) tyrosine phosphorylation levels (pY) in ^WT^ShcD‐expressing cells compared to control and mutant‐expressing cells in A (*n* = 4). (C) Stable MDA‐MB‐231BR cells were serum‐starved for 24 h, then seeded into Matrigel‐coated Boyden chambers and allowed to invade for 20 h (*n* = 3). (D) Schematic representation of the assembloid co‐culture experiment with an arrow highlighting cells from green fluorescent protein (GFP)‐labeled breast cancer spheroids infiltrating a TUJ1‐positive cerebral organoid. (E) Representative cross‐section images taken from the co‐culture between MDA‐MB‐231BR spheroids expressing FLAG, ShcD,^WT^ or ShcD^P*/S*^ and human cerebral organoids at day 17 showing the boundary of GFP‐positive breast cancer cells (green) infiltrating TUJ1‐positive organoids (magenta). Scale bar = 500 μm. Inset scale bar = 125 μm. (F) Quantification of assembloids at day 17 by measuring the percent co‐localization of GFP‐positive breast cancer cells and TUJ1‐positive organoid cells (*n* = 5 for FLAG‐alone, *n* = 8 for ShcD^WT^, and *n* = 3 for ShcD^P*/S*^). The data represent a minimum of three independent experiments; *P < 0.05; **P < 0.01. (B, C, F) *P*‐values represent significance levels from Kruskal‐Wallis ANOVA followed by Dunn's multiple comparison test. Data shown as mean ± SD.

The ability of ShcD to hijack EGFR signaling and promote cell invasion led us to next investigate whether these metastatic phenotypes would persist in a complex three‐dimensional (3D) model of tumor cell infiltration that we developed previously [15]. Since the brain is a common site for secondary tumor formation in metastatic breast cancers [59, 60], we used an iPSC system to generate human cerebral organoids [26] as an invasive substrate for a brain metastatic variant MDA‐MB‐231BR cell line [21]. To this end, we first generated stable FLAG, ShcD^WT^ and ShcD^P*/S*^ expressing MDA‐MB‐231BR cell lines and confirmed that the effects of ShcD on EGFR phosphorylation (Fig. 5A,B) and cell invasion (Fig. 5C) seen in MDA‐MB‐231 parental cells were conserved in this variant. Next, to model invasion in a 3D context, the stable MDA‐MB‐231BR cell lines were labeled with green fluorescent protein (GFP) and used to generate spheroids that were incubated with mature human cerebral organoids to form assembloids (Fig. 5D). Following 10 days of co‐culture, assembloids were frozen, cryosectioned, and immunostained for the neuronal marker TUJ1 to visualize organoid structure. Imaging revealed striking infiltration of ShcD^WT^‐expressing GFP‐positive cells toward the center of the organoid, while cells from spheroids expressing FLAG‐alone or ShcD^P*/S*^ appeared to remain at the edge of the organoid, with less infiltration (Fig. 5E). Quantification of MDA‐MB‐231BR cell infiltration across each group revealed an over 2‐fold increase with ShcD^WT^ compared to FLAG‐alone control and ShcD^P*/S*^ mutant (Fig. 5F). Overall, these findings further validate that ShcD phosphosignaling enhances breast cancer cell invasion, which may drive metastatic progression.

### 3.6 Indomethacin reduces EGFR–ShcD associations and ShcD‐mediated invasion in MDA‐MB‐231 cells

**Fig. 6:**
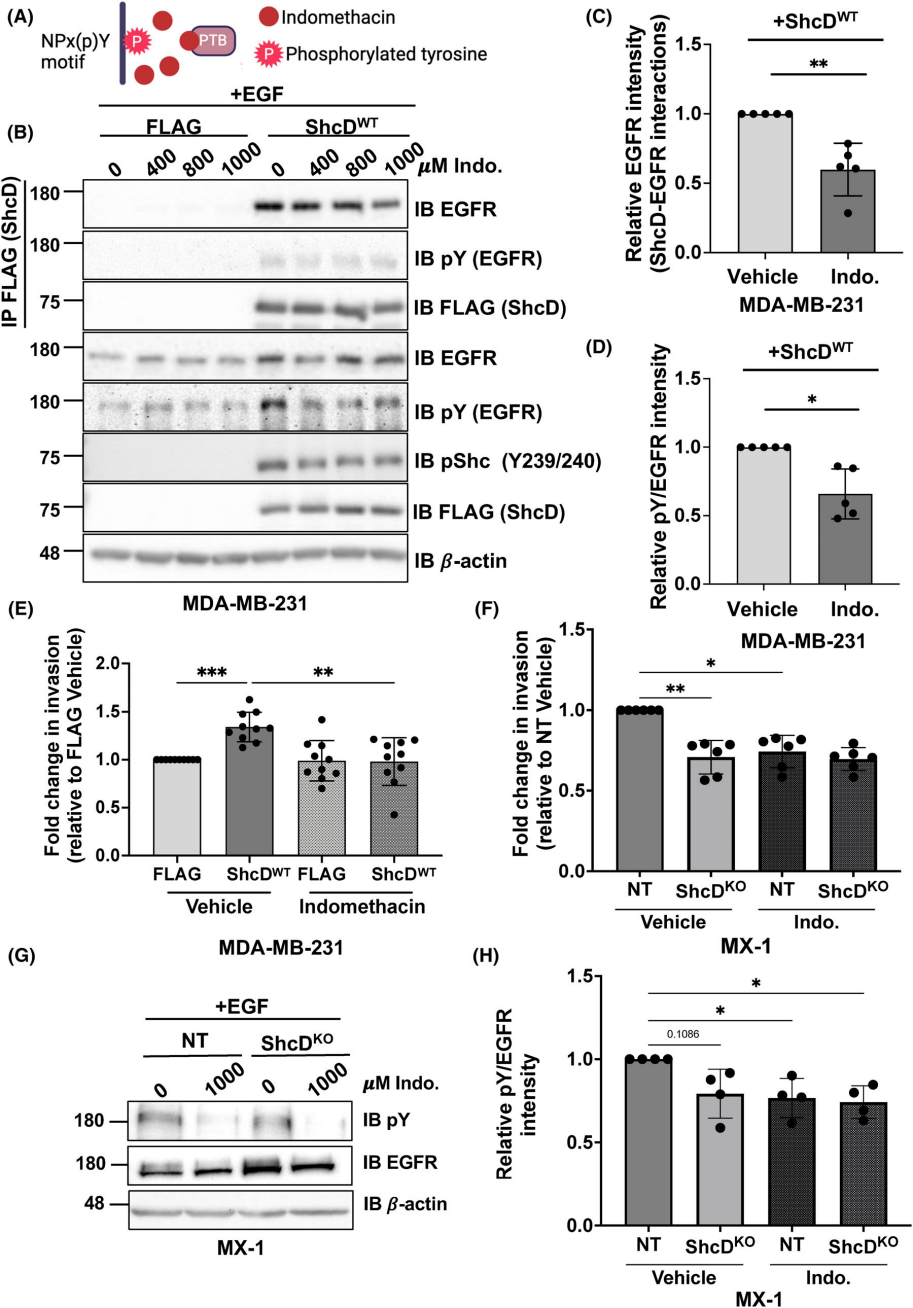
Indomethacin reduces ShcD‐EGFR associations and invasion in ShcD‐expressing breast cancer cells. (A) Indomethacin binds the Shc phosphotyrosine‐binding (PTB) domain to block interaction with phosphorylated NPXY motifs. (B) Stable MDA‐MB‐231 cells were treated with vehicle (dimethyl sulfoxide; DMSO) or the indicated concentrations of indomethacin (indo.) (400–1000 μm) for 24 h prior to stimulation with epidermal growth factor (EGF) (10 ng·mL^−1^; 10 min) and monitored for changes in levels of epidermal growth factor receptor (EGFR) and phosphotyrosine (pY) by immunoprecipitation (IP) and immunoblotting (IB) (*n* = 5). (C) Densitometric analysis of ShcD‐EGFR co‐immunoprecipitations in ShcD‐expressing cells treated with 1000 μm indomethacin compared to cells treated with vehicle in B (*n* = 5). (D) Densitometric analysis of EGFR tyrosine phosphorylation (pY) in ShcD‐expressing cells treated with 1000 μm indomethacin compared to cells treated with vehicle in B (*n* = 5). (E) Stable MDA‐MB‐231 cells expressing FLAG‐tagged wildtype ShcD (ShcD^WT^) or FLAG‐alone were pretreated with 1000 μm of indomethacin or vehicle (DMSO) for 24 h prior to seeding in Matrigel‐coated Boyden chambers for a 20 h period (*n* = 10). (F) Non‐targeting (NT) and ShcD knockout (KO) MX‐1 cells were treated with vehicle (DMSO) or 1000 μm indomethacin for 24 h prior to stimulation with EGF (10 ng·mL^−1^; 10 min) and profiled for changes in levels of EGFR and pY by immunoblotting (*n* = 4). (G) Densitometric analysis of EGFR tyrosine phosphorylation (pY) in NT and ShcD KO MX‐1 cells treated with 1000 μm indomethacin compared to cells treated with vehicle (*n* = 4). (H) NT and ShcD KO MX‐1 cells were pretreated with 1000 μm of indomethacin or vehicle (DMSO) for 24 h prior to seeding into Matrigel‐coated Boyden chambers and allowed to invade for 24 h (*n* = 6). The data represent a minimum of four independent experiments; *P < 0.05; **P < 0.01; ***P < 0.001. (C, D) *P*‐values represent significance levels from one‐sample *t*‐test. (E, G, H) *P*‐values represent significance levels from Kruskal‐Wallis ANOVA followed by Dunn's multiple comparison test. Data are shown as mean ± SD.

Expression of the ShcD^P*/S*^ mutant was able to disrupt EGFR binding and hyperphosphorylation as well as suppress invasion to levels comparable with control cells, leading us to hypothesize that specific targeting of ShcD–EGFR associations might have a similar effect. Indomethacin, a non‐steroidal anti‐inflammatory drug, binds to key amino acid residues (R67, K169, R175, F202) within the phosphotyrosine binding pocket of the ShcA PTB domain and competes with binding to NPx(p)Y motifs (such as those present on EGFR) [61] (Fig. 6A). We aligned sequences for ShcA and ShcD PTB domains to show the conservation of these four residues (Fig. S4A). We also confirmed that the PTB domain is the principal mode of contact between ShcD and EGFR in MDA‐MB‐231 cells, with minimal contribution from the SH2 domain, in line with our previous findings [14] (Fig. S4B).

We then evaluated the effect of indomethacin on ShcD–EGFR interactions. MDA‐MB‐231 FLAG‐alone and ShcD^WT^ stable cells were pretreated with increasing concentrations of indomethacin (0, 400, 800, or 1000 μm) prior to stimulation with EGF (Fig. 6B). Indomethacin progressively reduced ShcD‐EGFR associations, maximally at a concentration of 1000 μm, where there was a significant decrease in ShcD‐EGFR binding relative to vehicle‐treated cells (Fig. 6C). Simultaneously, we observed a significant decrease in EGFR phosphorylation in ShcD‐expressing cells treated with 1000 μm indomethacin compared to vehicle (Fig. 6D).

We next assessed the effects of this signaling inhibitor on ShcD‐induced cell invasion. Stable MDA‐MB‐231 cells expressing FLAG‐alone and ShcD^WT^ were pretreated for 24 h with 1000 μm indomethacin or vehicle and assayed for invasion through a Matrigel‐coated transwell where drug concentrations were maintained (Fig. 6E). As anticipated, in the absence of the drug, ShcD^WT^ cells displayed significantly enhanced invasion compared with FLAG‐alone control cells. However, the addition of indomethacin significantly reduced ShcD^WT^ cell invasion. Importantly, indomethacin appeared to have minimal influence on FLAG‐expressing cells, as both drug‐d vehicle‐treated cells displayed similar levels of invasion. We measured cell viability in parallel, and although slight differences existed in growth rates between treatment cohorts, no significant differences were observed within each treatment group (Fig. S5A).

Lastly, we tested the effect of indomethacin in MX‐1 cells, which express high levels of endogenous ShcD. In NT and ShcD KO MX‐1 cells, indomethacin similarly inhibited ShcD‐mediated invasion (Fig. 6F) with no significant effect on viability (Fig. S5D). We also treated parental MX‐1 cells with increasing concentrations of indomethacin (0 μm (vehicle), 400, 800, or 1000 μm) prior to stimulation with EGF and observed reduced EGFR tyrosine phosphorylation with all concentrations (Fig. S5B). Quantification of EGFR phosphorylation levels revealed a significant decrease between MX‐1 cells treated with 1000 μm indomethacin compared to those treated with vehicle (Fig. S5C). We likewise observed a decrease in EGFR phosphorylation in NT MX‐1 cells treated with 1000 μm indomethacin compared to ShcD KO cells and to NT cells treated with vehicle (Fig. 6G,H). Altogether, our results demonstrate that forced uncoupling of the ShcD PTB domain from EGFR using indomethacin reduces ShcD‐induced EGFR hyperphosphorylation and cell invasion in breast cancer cells.

## 4 Discussion

Herein, we have identified ShcD upregulation in TNBC and demonstrated that its expression enhances breast cancer cell invasion in two‐ and three‐dimensional settings. To our knowledge, this is the first report investigating brain‐tropic breast cancer cell infiltration into human cerebral organoids. Mechanistically, we defined the ShcD breast cancer interactome in MDA‐MB‐231 cells and uncovered novel associations between ShcD and components of the PI3K/Akt pathway. We also explored the ability of ShcD to facilitate signaling outcomes and showed that ShcD promotes heightened phosphorylation of EGFR while concurrently repressing Akt phosphorylation. Lastly, we demonstrated that these molecular and cellular effects could be reverted by disabling ShcD PTB domain function by mutation or pharmacological inhibition with indomethacin. Altogether, our data position ShcD as a candidate oncogene driving metastasis in TNBC.

In our pan‐cancer analysis, increased ShcD expression was also noted in brain (LGG and GBM) and skin (SKCM and UVM) origin cancers. High ShcD expression is associated with a poor outcome in melanoma [16, 17] and glioma [15] patients and has been recently linked to altered prostate cancer cell growth [62]. There have also been reports of a potential deleterious function for ShcD in liver [63, 64] and lung [65, 66, 67, 68] cancers. Using TCGA data via the TIMER2 portal [69, 70] (http://timer.cistrome.org/), we found a significant effect of high ShcD expression on decreased survival in advanced stages of breast (BRCA), lung (LUAD) and liver (LIHC) cancers (Fig. S6A), while the independent PRECOG database [71] (https://precog.stanford.edu/) associates high ShcD with lower survival in breast, colon, and ovarian cancers (Fig. S6B). ShcD is also upregulated in patients with vimentin‐positive invasive breast carcinoma of no special type, where it is associated with worse outcomes [72].

In support of ShcD as a bona fide target in breast cancer, this disease is the top predicted DisGeNet Annotation for ShcD in HumanNet [73] and multiple independent unbiased data analyses have identified ShcD as part of a prognostic signature in breast cancers [74, 75, 76] and in the Basal‐like/TNBC subtype specifically [77, 78, 79, 80]. Moreover, while this manuscript was in preparation, we note that others reported increased ShcD in TNBC tumors and cell lines [81, 82] as well as a positive correlation between ShcD expression and enhanced breast cancer cell invasion *in vitro* and metastasis *in vivo* [83], in line with our findings.

Through our unbiased AP‐MS screen, we identified both known and novel WT ShcD interactors in MDA‐MB‐231 cells. We speculate that this combination of interactors could represent a breast cancer‐specific ShcD interactome, as several have known functions in breast cancer, including BAG2 [84], GAB1/2 [85], MEMO1 [33], PEAK1 [86, 87], and SQSTM1 [88] or in other cancer types, such as ARAP1 [89], RFFL [90], OSMR [91], and ERFFI1 [92]. We also observed, consistent with the well‐characterized EGFR–ShcD interaction and a number of EGFR‐related GO terms (Table S2), that many of the proteins identified are known direct and indirect EGFR binding partners. For instance, of the 18 core EGFR/ShcA complex components identified in Wang *et al*. [42], 10 interact with ShcD after the addition of the previously characterized ShcD binding partner PTPN11/SHP2 [58] to the preys identified in our AP‐MS screen. Substituting ShcD for ShcA, this includes two complete sub‐complexes: the initial membrane‐bound EGFR complex and a newly identified receptor‐free complex (ShcA/ShcD, GRB2, ARHGEF5, GAREM1 and LRRK1). In support of our findings, an interaction between ShcD and LRRK1 in HCT116 colon cancer cells was recently identified by the BioPlex [35] project.

We recovered a smaller set of 19 interactors with ShcD^P*/S*^. As anticipated, known PTB/SH2 dependent binding partners such as EGFR and Grb2 were not recovered with the mutant protein. However, perhaps somewhat unexpectedly, 10 of the interactors also bound ShcD^WT^. These interactors shared between WT and P*/S* mutant ShcD must not be reliant on ShcD recruitment to phosphorylated tyrosine residues such as those on EGFR and other receptors. Instead, alternative types of ShcD protein–protein interactions could include phosphotyrosine‐independent SH2 domain [93, 94] and PTB domain [95, 96] binding modes, as well as recruitment of interacting proteins to phosphorylated tyrosines, serines, or proline‐rich motifs present throughout the protein.

The ability of ShcD to promote heightened EGFR phosphorylation is a unique function within the Shc family. Several reports show that PTB and SH2 domains can provide phosphoprotection to target receptors by binding phosphotyrosine sites and blocking phosphatase‐driven dephosphorylation [14, 97]. Given the nature of ShcD as an adapter protein, it is reasonable to believe that ShcD could promote EGFR phosphorylation through a similar mechanism. These findings are intriguing when considering the correlation between EGFR hyperphosphorylation and breast cancer progression. EGFR hyperactivation is common in triple‐negative tumors despite a low frequency of EGFR activating mutations [98], indicating other mechanisms are driving aberrant EGFR signaling. Since heightened EGFR activity promotes breast cancer cell invasion, it is plausible that ShcD‐induced EGFR hyperphosphorylation may contribute to metastatic phenotypes in breast cancer.

Our model of ShcD overexpression results in a seemingly counterintuitive simultaneous hyperphosphorylation of EGFR and reduction in Akt activation. This was evident in our enriched GO terms, which included both “EGFR signaling” and “Negative regulation of PI3K/AKT.” There could be several potential explanations for these observations. The particular p85 subunit (PI3KR1) identified in our AP‐MS screen tends to be inhibitory to Akt activation [97]. As ShcD binds both EGFR and p85, ShcD may function to recruit this inhibitory p85 isoform to active EGFR‐GAB1‐PI3K complexes, facilitating PI3K inhibition rather than activation. Alternatively, ShcD may hinder localization of Akt, thus preventing its phosphorylation, analogous to what has been observed in melanoma, where ShcD can mislocalize DOCK4 protein and alter RAC1 signaling [17]. Supporting our findings, in their initial characterization of ShcD, Fagiani *et al* observed that ShcD/RaLP overexpression does not activate Akt and in fact may limit the pathway in melanoma [16]. Indeed, while conventionally considered drivers of tumourigenesis, accumulating reports suggest that, under some circumstances, Akt proteins can function as suppressors of invasion and metastasis [4, 5, 6].

We demonstrate that, in a two‐dimensional system (2D), ShcD expression increases invasion and decreases the capacity of breast cancer cells to adhere to an extracellular matrix, suggesting that ShcD may promote the detachment of cancer cells from the surrounding microenvironment *in vivo* to escape the primary tumor site during metastasis. Our findings are in line with other reports that show ShcD silencing in melanoma cells enhances adhesion and reduces invasion [16, 17]. The ability of ShcD to enhance cancer cell invasion is further corroborated herein using a 3D assembloid model where ShcD expression promotes infiltration of brain metastatic breast cancer‐derived spheroids into human cerebral organoids. Intriguingly, increased ShcD levels were detected in 3D vs. 2D cell culture models of endothelial hemangiomas [99]. The incidence of brain metastases in patients with TNBC has been shown to be as high as 46% [100], and a recent report suggests ShcD is one of a handful of genes highly expressed in brain metastasized breast cancers [101].

**Fig. 7:**
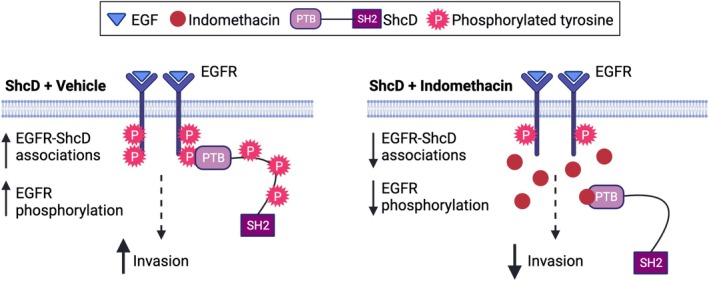
Schematic showing the effect of indomethacin in reducing ShcD‐mediated EGFR hyperphosphorylation and invasion. In untreated cells (left), ShcD binds the epidermal growth factor receptor (EGFR) via its phosphotyrosine‐binding (PTB) domain and confers heightened receptor phosphorylation and increased invasion. In the presence of indomethacin, which occupies the PTB domain (right), ShcD‐EGFR interactions decrease concomitant with a reduction in EGFR phosphorylation and breast cancer cell invasion.

Aberrant EGFR activation is a common hallmark of breast cancer, and while several anti‐EGFR therapies have been developed, triple‐negative tumors appear clinically resistant or unresponsive [102]. Interestingly, the ShcD interactome aligns with tyrosine kinase inhibitor resistance, supporting an avenue to target protein–protein interactions rather than individual RTKs in these tumors [103, 104, 105]. Indeed, in recent years, such small molecule inhibitors have demonstrated potential for the treatment of cancer [106, 107] and have even reached clinical trials [108]. Accordingly, indomethacin has shown promising anticancer properties *in vitro*, such as the ability to inhibit cancer cell proliferation, epithelial‐to‐mesenchymal transition, and invasion [109, 110, 111, 112]. Given the recent observation that it binds the PTB domain of ShcA [61], we introduced indomethacin to MDA‐MB‐231 cells overexpressing ShcD and disrupted EGFR‐ShcD complexes with an associated decrease in EGFR phosphorylation and cell invasion. A similar decrease in EGFR phosphorylation after indomethacin treatment was observed in MX‐1 cells with high endogenous ShcD expression, confirming that EGFR hyperphosphorylation is reversible, per our proposed mechanism (Fig. 7). Notably, the effect of indomethacin did not change following ShcD KO in these cells, supporting the notion that other Shc paralogs may not be compensating in this setting, and in line with the unique signaling phenotype of ShcD compared to canonical ShcA signaling in breast cancer progression [45, 46]. As recent trends in drug development aim to repurpose existing approved compounds for novel applications [113, 114, 115], research into the use of indomethacin in cancer treatment warrants further investigation.

## 5 Conclusions

In summary, our data demonstrate that ShcD drives pro‐metastatic outcomes in breast cancer cells, and it supports a basis for selective inhibition of RTK/adaptor complexes in suppressing the invasive phenotype of cancer cells. ShcD thus represents a novel prognostic and therapeutic target in TNBC.

## Conflict of interest

Dr. Jones received funding from the Cancer Research Society and CIHR. Drs. Bisson, Gingras, and Jones received funding from the Canada Research Chairs program. The remaining authors declare no competing interests.

## Author contributions

HRL and NJ conceived the study; HRL, BA, and HSS performed experiments; BA and JL established the assembloid experiments; MT and LAN performed database analysis; SLB and CEM performed AP‐MS experiments and AP‐MS analysis; ACG and NB provided instrument access and reviewed AP‐MS analysis results; HRL drafted the initial version of the manuscript with assistance from HNR and HSS; HRL, HSS, HNR, and LAN prepared figures with assistance from CEM; HSS, LAN, and NJ revised the manuscript; all authors reviewed the final manuscript.

## Peer review

The peer review history for this article is available at https://www.webofscience.com/api/gateway/wos/peer‐review/10.1002/1878‐0261.70022.

## Supporting information


**Fig. S1.** *In silico* analysis of ShcD expression in breast cancer and breast cancer cell lines. (A) University of Alabama at Birmingham MammOnc‐DB [50] analysis of *SHC4* expression in METABRIC [48] samples by Triple Negative (TNBC) status. *P < 0.05 calculated by MammOnc‐DB. Accessed October 2024 at http://resource.path.uab.edu/cgi‐bin1/MammOnc‐gene‐Result.pl?genenam=SHC4&ctype=METABRIC. Box and whisker bars indicate the minimum and maximum values. (B) University of Alabama at Birmingham MammOnc‐DB [50] analysis of *SHC4* expression in SCAN‐B [49] samples by Triple Negative (TNBC) status. *P < 0.05 calculated by MammOnc‐DB. Accessed October 2024 at http://resource.path.uab.edu/cgi‐bin1/MammOnc‐gene‐Result.pl?genenam=SHC4&ctype=SCANB. Box and whisker bars indicate the minimum and maximum values. (C) GENT2 database [51] analysis of *SHC4* expression in breast cancer by subtype. Accessed October 2024 at http://gent2.appex.kr/gent2/. Subtypes with less than 100 samples are not shown. Box and whisker bars indicate the minimum and maximum values within 1.5 times the inter‐quartile range. (D) Kmplot.com [52] analysis of *SHC4* expression on overall survival of lymph node positive patients (*n* = 814) from all breast cancer subtypes. Accessed May 2023. (E) Single cell breast cancer atlas [55] (https://bcatlas.tigem.it) UMAP expression profile of *SHC4* across 32 representative breast cancer cell lines. Accessed January 2024. H, HER2; LA, Luminal A; LB, Luminal B; TNA, Triple Negative A; TNB, Triple Negative B.

**Fig. S2.** Analysis of ShcD overexpressing MCF7 cells. (A) MCF‐7 cells stably expressing FLAG‐alone or ShcD‐FLAG were generated and verified for expression by immunoblot (IB). (B) Stable MCF‐7 cells were serum starved for 24 h and seeded into Matrigel‐coated chambers and allowed to invade for 24 h (*n* = 4). (C) Cell viability was measured in parallel (*n* = 4). *P < 0.05. *P*‐values represent significance levels from repeated measures two‐way ANOVA. Data shown as mean ± SD.

**Fig. S3.** Confirmation of successful CRISPR deletion of SHC4 in MX‐1 cells. (A) Location of CRISPR guide targeting Exon 1 of ShcD/*SHC4*. PAM sequence is underlined. Protein structure generated using cBioportal Mutation Mapper (http://www.cbioportal.org/mutation_mapper) with modified labels added in Inkscape. (B) Tracking of Indel Decomposition (TIDE) analysis [25] indicates 78.9% CRISPR guide target efficiency for SHC4 gene editing. TIDE analysis accessed via http://shinyapps.datacurators.nl/tide.

**Fig. S4.** Investigating binding properties of the ShcD PTB domain. (A) Multiple sequence alignment (Clustal Omega Align tool at https://www.uniprot.org/align) of p52 ShcA (Uniprot: P29353‐2) and ShcD (Uniprot: Q6S5L8) showing that key amino acids in the phosphotyrosine binding pocket of the PTB domain that mediate indomethacin binding to ShcA (R67, A175, K169, F202) [62] are conserved in ShcD. (B) FLAG immunoprecipitants from MDA‐MB‐231 cells transiently expressing FLAG‐tagged wildtype ShcD (ShcD^WT^), PTB* mutant ShcD (ShcD^PTB*^) or SH2* mutant ShcD (ShcD^SH2*^) domain mutants were probed for the indicated antibodies by immunoblotting (IB) (*n* = 1).

**Fig. S5.** Indomethacin alters EGFR phosphorylation in ShcD‐expressing breast cancer cells. (A) MDA‐MB‐231 cell viability was measured in parallel with transwell invasion assay in Fig. 6E to ensure similar growth rates across each treatment group (*n* = 10). (B, C) MX‐1 parental cells (B) were treated with vehicle (dimethyl sulfoxide; DMSO) or the indicated concentrations of indomethacin (indo.; 400‐1000 μm) for 24 h prior to stimulation with epidermal growth factor (EGF) (10 ng·mL^−1^; 10 min) and profiled for changes in levels of epidermal growth factor receptor (EGFR) and tyrosine phosphorylation (pY) by immunoblotting (IB), followed by (C) Densitometric analysis of EGFR tyrosine phosphorylation (pY) in MX‐1 cells treated with 1000 μm indomethacin compared to cells treated with vehicle (*n* = 5). Data shown as mean ± SD. (D) MX‐1 cell viability was measured in parallel with transwell invasion assay in Fig. 6H to ensure similar growth rates across each treatment group (*n* = 6). The data represent a minimum of 5 independent experiments. **P < 0.01. (A, D) *P*‐values represent significance from repeated measures two‐way ANOVA. (C) *P*‐values represent significance levels from one sample *t*‐test. Data shown as mean ± SD.

**Fig. S6.** Multi‐cancer analysis of ShcD expression levels on overall survival. (A) Forest Plot (Hazard ratios and 95% confidence intervals are indicated) of Timer 2.0 [70] (http://timer.cistrome.org) analysis of contribution of *SHC4* expression to overall survival in TCGA datasets for breast (BRCA), lung (LUAD) and liver (LIHC) cancers, by Stage. Graph generated using HiPlot [71] (https://hiplot.cn/basic/metawho and https://hiplot.cn/basic/custom‐heat‐map) and assembled in Photoshop and Inkscape. (B) Precog [72] (https://precog.stanford.edu) Z‐scores for *SHC4* for all cancer types with a Z‐score > 0. A higher Z‐score indicates increased gene expression has a negative effect on overall survival. Graph generated using Graphpad Prism.

**Table S1.** Summary of DIA‐NN analysis of all preys detected by AP‐MS with ShcD^WT^ and ShcD^P*/S*^ baits as compared to control (FLAG) samples.

**Table S2.** gProfiler analysis of ShcD^WT^ enriched AP‐MS preys.

## Data Availability

Summarized AP‐MS data is available in supplemental data; raw data is available on request. The following publicly available datasets were used: E‐MTAB‐2770. Cancer expression data, as detailed in the Methods, was retrieved from TCGA (https://www.cancer.gov/tcga). Other publicly available data was analyzed using online platforms, as described in the Methods.
