# Supplementary figures and images for: ShcD adaptor protein drives invasion of triple negative breast cancer cells by aberrant activation of EGFR signaling
Source: Mol Oncol. 2025 Mar 28;19(10):2833–59. doi: 10.1002/1878-0261.70022 (PMC12515706; doi:10.1002/1878-0261.70022)

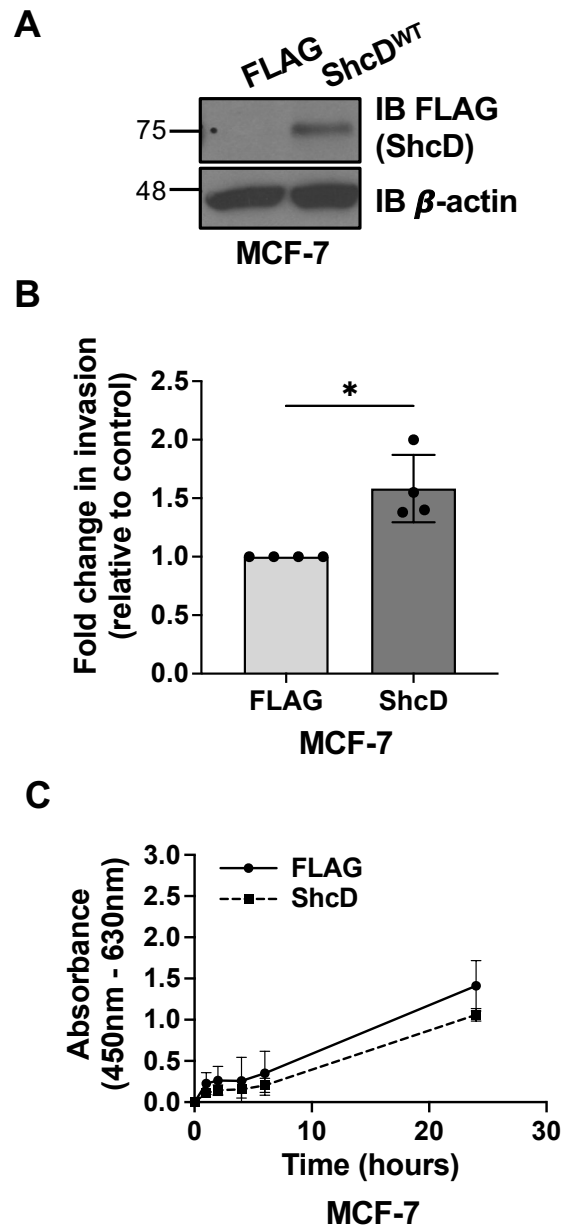

Supplement: Supplementary file 2 — Fig. S2. Analysis of ShcD overexpressing MCF7 cells. (A) MCF‐7 cells stably expressing FLAG‐alone or ShcD‐FLAG were generated and verified for expression by immunoblot (IB). (B) Stable MCF‐7 cells were serum starved for 24 h and seeded into Matrigel‐coated chambers and allowed to invade for 24 h (n = 4). (C) Cell viability was measured in parallel (n = 4). *P < 0.05. P‐values represent significance levels from repeated measures two‐way ANOVA. Data shown as mean ± SD. [file MOL2-19-2833-s006.pdf]

## A Crispr Guide Location

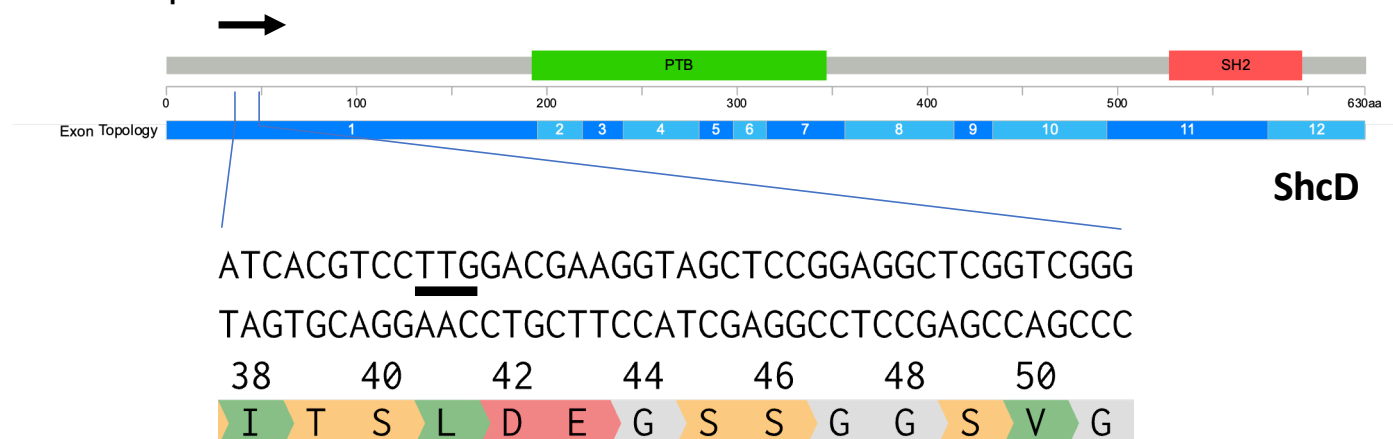

## B

## TIDE Analysis Results

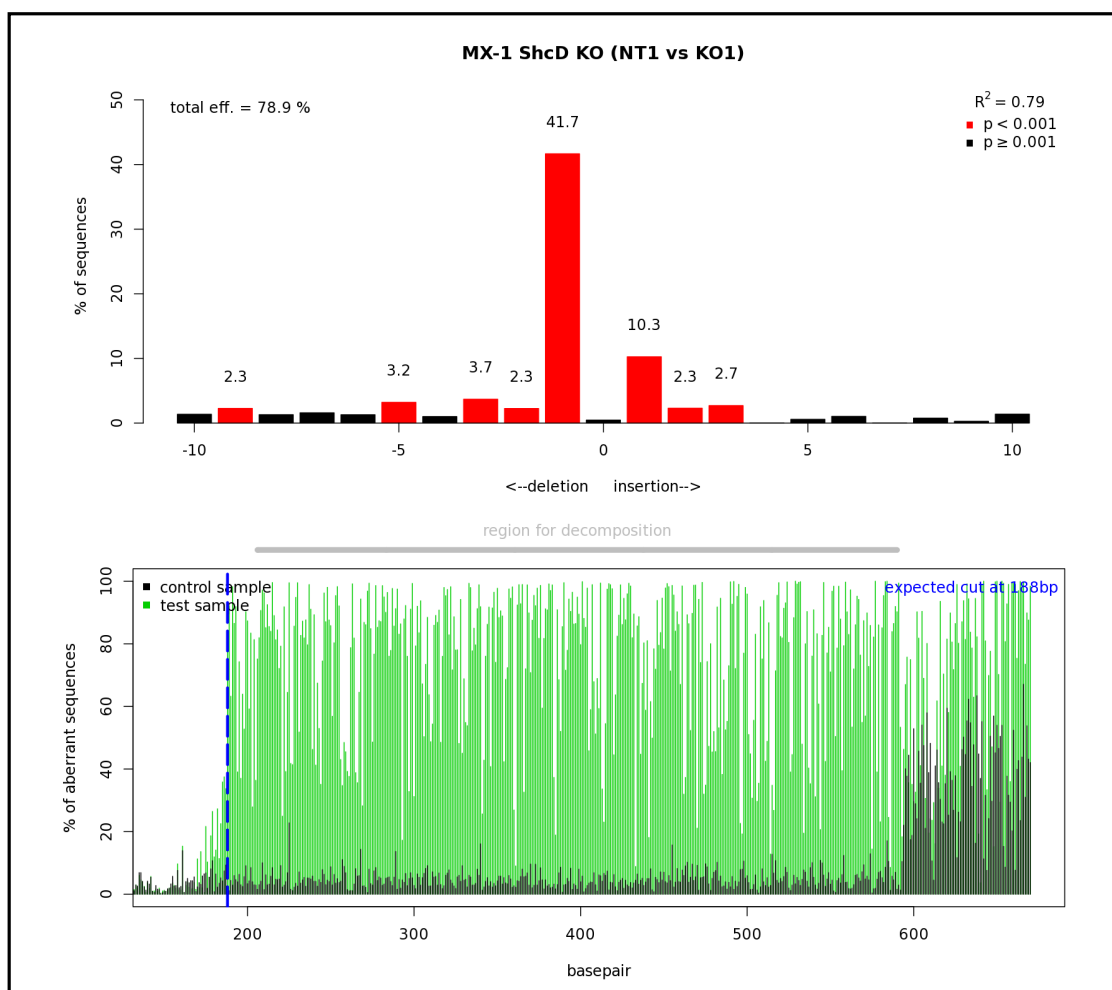

Supplement: Supplementary file 3 — Fig. S3. Confirmation of successful CRISPR deletion of SHC4 in MX‐1 cells. (A) Location of CRISPR guide targeting Exon 1 of ShcD/SHC4. PAM sequence is underlined. Protein structure generated using cBioportal Mutation Mapper (http://www.cbioportal.org/mutation_mapper) with modified labels added in Inkscape. (B) Tracking of Indel Decomposition (TIDE) analysis [25] indicates 78.9% CRISPR guide target efficiency for SHC4 gene editing. TIDE analysis accessed via http://shinyapps.datacurators.nl/tide. [file MOL2-19-2833-s001.pdf]

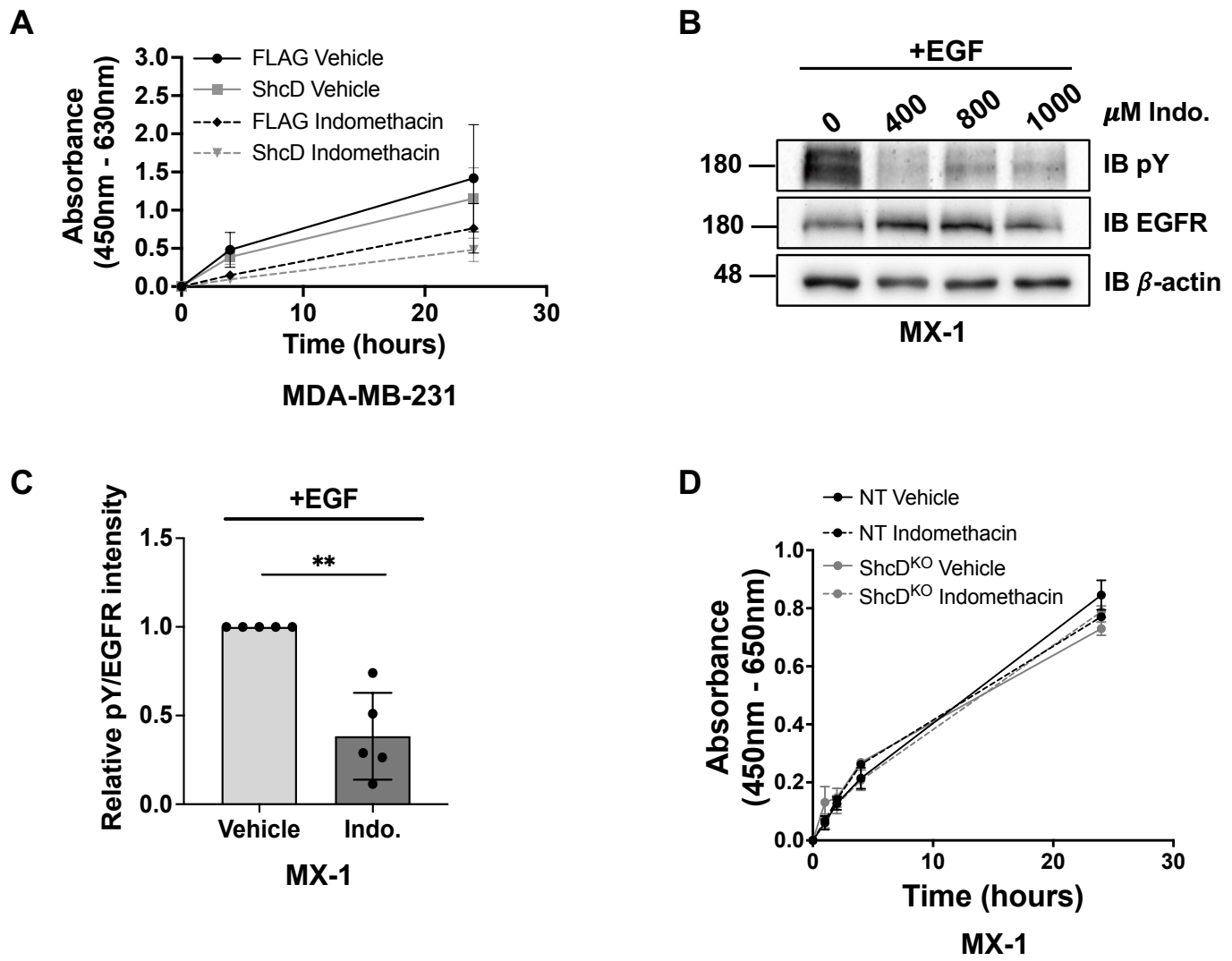

Supplement: Supplementary file 5 — Fig. S5. Indomethacin alters EGFR phosphorylation in ShcD‐expressing breast cancer cells. (A) MDA‐MB‐231 cell viability was measured in parallel with transwell invasion assay in Fig. 6E to ensure similar growth rates across each treatment group (n = 10). (B, C) MX‐1 parental cells (B) were treated with vehicle (dimethyl sulfoxide; DMSO) or the indicated concentrations of indomethacin (indo.; 400‐1000 μm) for 24 h prior to stimulation with epidermal growth factor (EGF) (10 ng·mL−1; 10 min) and profiled for changes in levels of epidermal growth factor receptor (EGFR) and tyrosine phosphorylation (pY) by immunoblotting (IB), followed by (C) Densitometric analysis of EGFR tyrosine phosphorylation (pY) in MX‐1 cells treated with 1000 μm indomethacin compared to cells treated with vehicle (n = 5). Data shown as mean ± SD. (D) MX‐1 cell viability was measured in parallel with transwell invasion assay in Fig. 6H to ensure similar growth rates across each treatment group (n = 6). The data represent a minimum of 5 independent experiments. **P < 0.01. (A, D) P‐values represent significance from repeated measures two‐way ANOVA. (C) P‐values represent significance levels from one sample t‐test. Data shown as mean ± SD. [file MOL2-19-2833-s007.pdf]

A

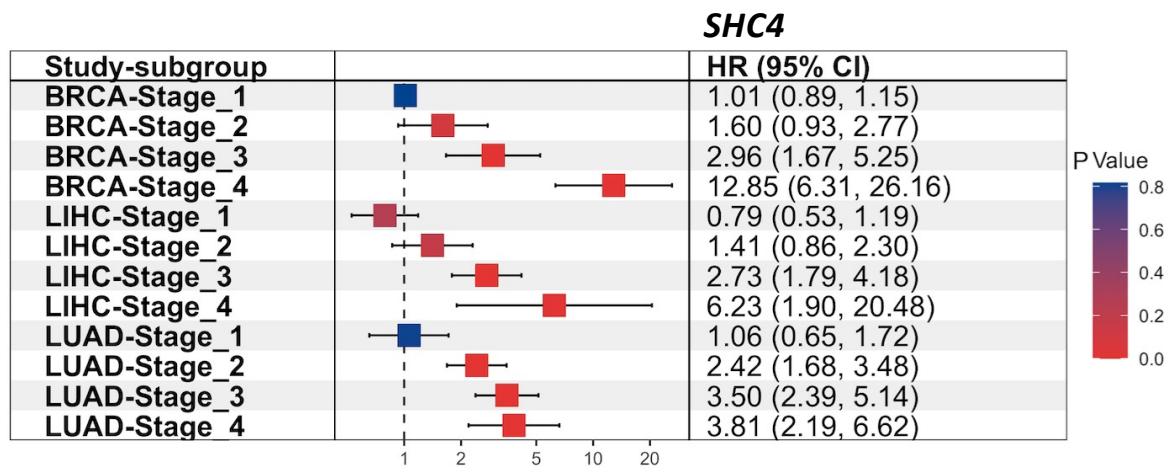

B

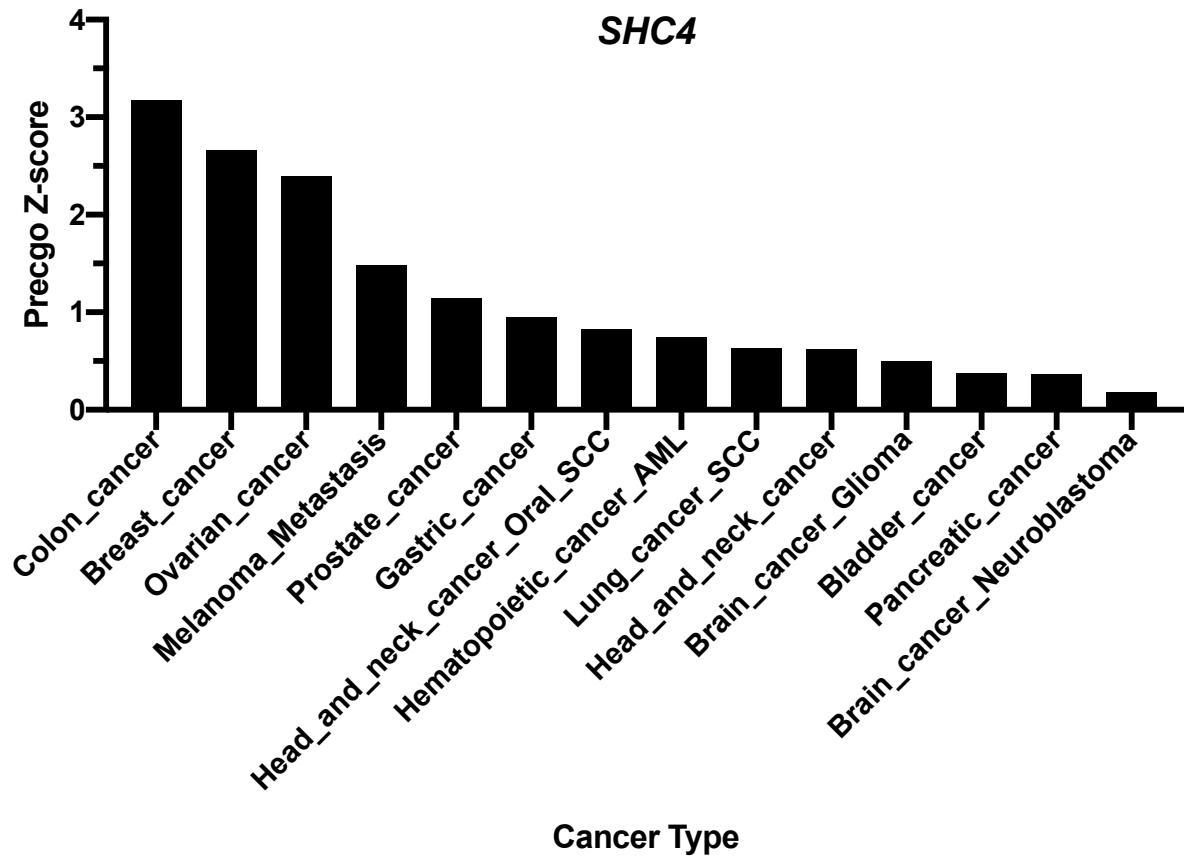

Supplement: Supplementary file 6 — Fig. S6. Multi‐cancer analysis of ShcD expression levels on overall survival. (A) Forest Plot (Hazard ratios and 95% confidence intervals are indicated) of Timer 2.0 [70] (http://timer.cistrome.org) analysis of contribution of SHC4 expression to overall survival in TCGA datasets for breast (BRCA), lung (LUAD) and liver (LIHC) cancers, by Stage. Graph generated using HiPlot [71] (https://hiplot.cn/basic/metawho and https://hiplot.cn/basic/custom‐heat‐map) and assembled in Photoshop and Inkscape. (B) Precog [72] (https://precog.stanford.edu) Z‐scores for SHC4 for all cancer types with a Z‐score > 0. A higher Z‐score indicates increased gene expression has a negative effect on overall survival. Graph generated using Graphpad Prism. [file MOL2-19-2833-s005.pdf]
