# Supplementary material for: ShcD adaptor protein drives invasion of triple negative breast cancer cells by aberrant activation of EGFR signaling
Source: Mol Oncol. 2025 Mar 28;19(10):2833–59. doi: 10.1002/1878-0261.70022 (PMC12515706; doi:10.1002/1878-0261.70022)
Supplement: Supplementary file 4 — Fig. S4. Investigating binding properties of the ShcD PTB domain. (A) Multiple sequence alignment (Clustal Omega Align tool at https://www.uniprot.org/align) of p52 ShcA (Uniprot: P29353‐2) and ShcD (Uniprot: Q6S5L8) showing that key amino acids in the phosphotyrosine binding pocket of the PTB domain that mediate indomethacin binding to ShcA (R67, A175, K169, F202) [62] are conserved in ShcD. (B) FLAG immunoprecipitants from MDA‐MB‐231 cells transiently expressing FLAG‐tagged wildtype ShcD (ShcDWT), PTB* mutant ShcD (ShcDPTB*) or SH2* mutant ShcD (ShcDSH2*) domain mutants were probed for the indicated antibodies by immunoblotting (IB) (n = 1). [file MOL2-19-2833-s008.pdf]

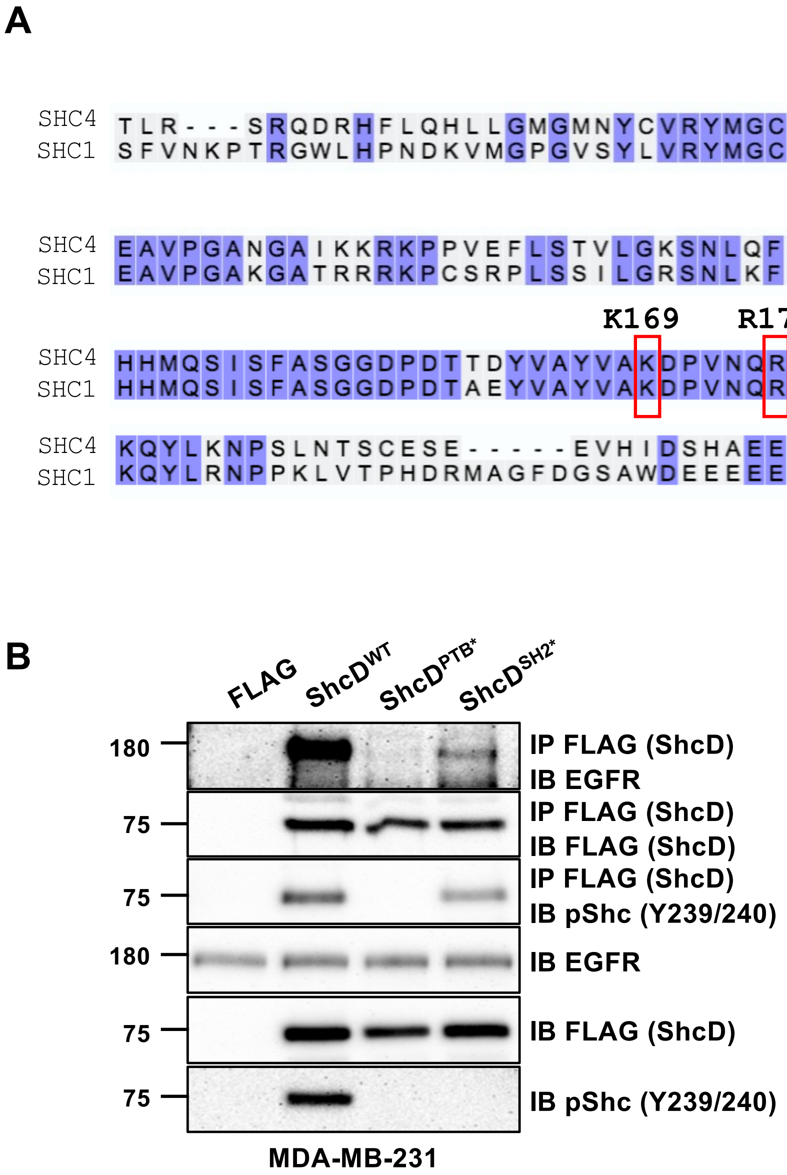

Supp Figure 4
