# Supplementary material for: ShcD adaptor protein drives invasion of triple negative breast cancer cells by aberrant activation of EGFR signaling
Source: Mol Oncol. 2025 Mar 28;19(10):2833–59. doi: 10.1002/1878-0261.70022 (PMC12515706; doi:10.1002/1878-0261.70022)
Supplement: Supplementary file 1 — Fig. S1. In silico analysis of ShcD expression in breast cancer and breast cancer cell lines. (A) University of Alabama at Birmingham MammOnc‐DB [50] analysis of SHC4 expression in METABRIC [48] samples by Triple Negative (TNBC) status. *P < 0.05 calculated by MammOnc‐DB. Accessed October 2024 at http://resource.path.uab.edu/cgi‐bin1/MammOnc‐gene‐Result.pl?genenam=SHC4&ctype=METABRIC. Box and whisker bars indicate the minimum and maximum values. (B) University of Alabama at Birmingham MammOnc‐DB [50] analysis of SHC4 expression in SCAN‐B [49] samples by Triple Negative (TNBC) status. *P < 0.05 calculated by MammOnc‐DB. Accessed October 2024 at http://resource.path.uab.edu/cgi‐bin1/MammOnc‐gene‐Result.pl?genenam=SHC4&ctype=SCANB. Box and whisker bars indicate the minimum and maximum values. (C) GENT2 database [51] analysis of SHC4 expression in breast cancer by subtype. Accessed October 2024 at http://gent2.appex.kr/gent2/. Subtypes with less than 100 samples are not shown. Box and whisker bars indicate the minimum and maximum values within 1.5 times the inter‐quartile range. (D) Kmplot.com [52] analysis of SHC4 expression on overall survival of lymph node positive patients (n = 814) from all breast cancer subtypes. Accessed May 2023. (E) Single cell breast cancer atlas [55] (https://bcatlas.tigem.it) UMAP expression profile of SHC4 across 32 representative breast cancer cell lines. Accessed January 2024. H, HER2; LA, Luminal A; LB, Luminal B; TNA, Triple Negative A; TNB, Triple Negative B. [file MOL2-19-2833-s004.pdf]

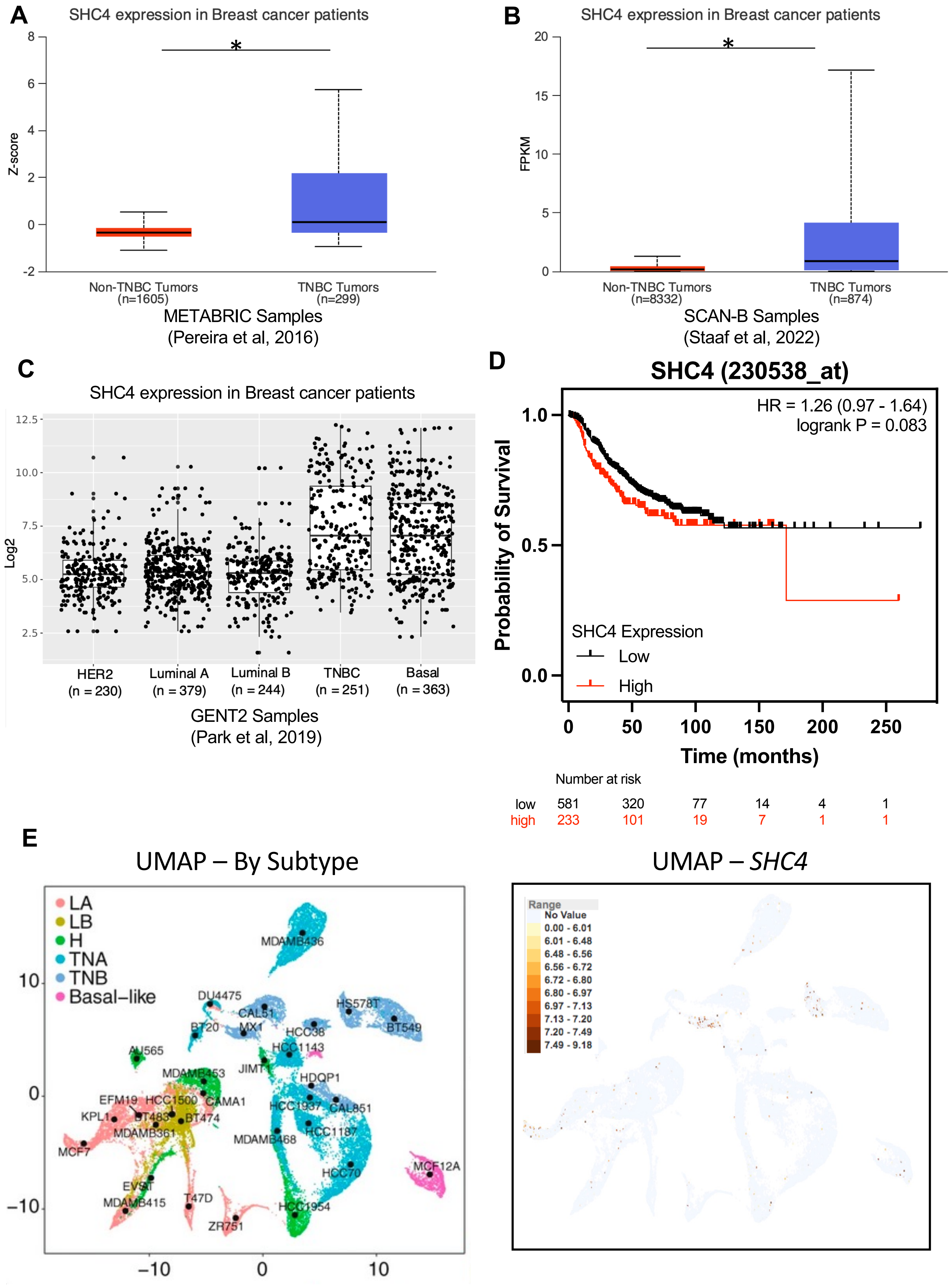

Supp Figure 1
